# A Lambda Red Recombinase‐Assisted CRISPR/Cas9 System for Efficient and Precise Genome Editing in *Agrobacterium tumefaciens*

**DOI:** 10.1111/pbi.70768

**Published:** 2026-09-30

**Authors:** Minmin Zheng, Rui Liu, Wei‐Chang Huang, Zuren Li, Kai Hua

**Affiliations:** ^1^ Shanghai Key Laboratory of Plant Functional Genomics and Resources, Shanghai Chenshan Botanical Garden, and Chenshan Science Research Center, CAS Center for Excellence in Molecular Plant Sciences (CEMPS) Chinese Academy of Sciences (CAS) Shanghai China; ^2^ Eastern China Conservation Centre for Wild Endangered Plant Resources Shanghai Chenshan Botanical Garden Shanghai China; ^3^ Yuelushan Laboratory Changsha China

**Keywords:** *Agrobacterium tumefaciens*, CRISPR/Cas9, lambada red recombinase, precise editing, strain engineering

## Abstract

*Agrobacterium tumefaciens* is a powerful delivery tool for plant genetic transformation and a pivotal platform for agricultural biotechnology. However, laboratory strains of *Agrobacterium* have remained genetically unoptimized for decades, with genome engineering hindered by inefficient, endogenous homologous recombination systems. Here, we developed and optimized a lambda (λ) Red recombinase‐assisted CRISPR/Cas9 system that enables efficient and precise introduction of point mutations, insertions, deletions and large fragment replacements into both the chromosome and Ti plasmids of different *A. tumefaciens* strains, with minimal off‐target editing. Editing efficiencies across eighteen target sites ranged from 26.7% to 100%, demonstrating the versatility of this method. Furthermore, we incorporated a *SacB* counter‐selection marker into our system that facilitates the plasmids curing after editing. Using this platform, we sequentially deleted four antibiotic‐resistance loci in strain EHA105, generating an antibiotic‐sensitive derivative, EHA105‐S, that maintains wild‐type growth kinetics and transformation efficiency. Additionally, we engineered the microbe‐associated molecular pattern (MAMPs) of two elongation factor Tu (EF‐Tu) genes, allowing the *A. tumefaciens* to escape EF‐Tu receptor (EFR) perception. The resulting engineered strains displayed enhanced transient expression efficiency in *Arabidopsis thaliana* . Our λ Red‐assisted CRISPR/Cas9 system thus provides a robust, scalable and user‐friendly tool for *Agrobacterium* genome engineering, paving the way for the design of next‐generation strains to revolutionize plant transformation and advance synthetic biology applications in plants.

## 1 Introduction

*Agrobacterium tumefaciens* is a Gram‐negative phytopathogen that causes crown gall disease on a wide range of dicotyledonous plants. During infection, *A. tumefaciens* transfers a segment of DNA known as transfer DNA (T‐DNA) from its tumour‐inducing (Ti) plasmid into plant cells, where it stably integrates into the host genome (Gelvin 2017). The T‐DNA region carries genes that direct the synthesis of plant hormone auxin and cytokinin, leading to uncontrolled cell proliferation and tumour formation at infection sites. This remarkable inter‐kingdom DNA transfer capability is mediated by the *vir* region of the Ti plasmid, which encodes the components of a Type IV secretion system (T4SS) (Gelvin 2012). The T4SS forms a molecular bridge between the bacterial and plant cell membranes, enabling the translocation of T‐DNA and some virulence effectors into the host (Lacroix and Citovsky 2019). Following the elucidation of molecular mechanisms of T‐DNA transfer, *A. tumefaciens* has been repurposed as a premier tool for plant and fungi genetic transformation. However, despite its central role in plant biotechnology, laboratory strains of *A. tumefaciens* have undergone minimal genetic optimization in the past four decades (De Saeger et al. 2021).

Traditional genome engineering in *A. tumefaciens* primarily relied on allelic replacement or transposon mutagenesis to generate optimized strains for plant transformation (Goralogia et al. 2025). The classic allelic replacement strategy employed double‐crossover homologous recombination, requiring two sequential selection steps (Lazo et al. 1991). In the first step, single crossover integrants were selected using an antibiotic resistance marker. In the second step, the *SacB* gene served as a counter‐selection marker to isolate edited clones carrying double‐crossover events. While effective, this method is slow, labour‐intensive and yields low recovery of desired clones due to the bacterium's inherently low efficiency of homologous recombination. Alternatively, transposon‐mediated insertional mutagenesis enables random gene disruption but demands screening of hundreds to thousands of colonies to identify insertions within a specific target gene, a process both inefficient and impractical for multiplex or precise editing (Klapwijk et al. 1980).

The advent of CRISPR/Cas9‐based tools has revolutionized bacterial genome engineering (Zhao et al. 2020). Rodrigues et al. recently developed a cytosine base editor in *Agrobacterium* that enables efficient gene inactivation by introducing premature stop codons (Rodrigues et al. 2021). However, base editing is constrained by the types of mutations it can induce, stringent PAM sequence requirements and potential sgRNA‐independent off‐target effects. More recently, an RNA‐guided DNA integration system has been developed for genome engineering in *A. tumefaciens* (Aliu et al. 2022). The INTEGRATE system (INsertion of Transposable Elements by Guide RNA–Assisted TargEting) combines the TniQ‐Cascade complex for RNA‐guided DNA targeting with transposase proteins TnsA, TnsB and TnsC to catalyse precise, directional insertion of cargo DNA into genomic target sites, enabling targeted gene disruption with no detectable off‐target effects. When combined with the Cre‐*loxP* recombination system, the INTEGRATE system can also mediate large fragment deletions (Aliu et al. 2022). Nonetheless, the INTEGRATE system leaves behind a residual scar after editing, and previously integrated cargo elements may be remobilized upon reuse of the same system, compromising its utility for sequential editing. Therefore, there remains a strong need for a rapid, easy‐to‐use and scarless genome editing platform in *A. tumefaciens* capable of introducing diverse mutation types.

Recombineering, a powerful genome engineering strategy derived from bacteriophage‐encoded homologous recombination systems, has become a widely adopted tool for precise bacterial genome modification (Corts et al. 2022). One of best‐characterized recombineering systems is the λ Red system from phage λ, which encodes a 5′‐3′ exonuclease Redα, an ssDNA‐annealing protein Redβ and a Gam protein Redγ. Redα processes the linear dsDNA substrates to produce 3′ single‐stranded overhangs, which are then protected and annealed to complementary ssDNA by Redβ. Concurrently, Redγ inhibits the host RecBCD exonuclease, preventing the degradation of exogenous linear DNA and thereby enhancing recombination efficiency (Datsenko and Wanner 2000). Through this coordinated action, the λ Red system enables genome modification using linear double‐ or single‐stranded DNA donors with relatively short homologous arms. The system has been extensively applied for genome engineering in *E. coli* and closely related bacteria, including *A. tumefaciens* (Corts et al. 2022; Hu et al. 2014). However, in the absence of selection, recombination efficiencies remain low, making isolating of desired mutants labour‐intensive and inefficient. To overcome this limitation, the λ Red system has been successfully coupled with CRISPR/Cas9 system to enrich the editing events (Jiang et al. 2013; Pyne et al. 2015). The CRISPR/Cas9 mediated chromosome cleavage is lethal to most bacteria and can be used to counter‐select against unedited cell (Jiang et al. 2013). By introducing a lethal double‐strand break (DSB) into the unedited allele, only cells that have undergone successful editing can survive. This CRISPR‐Cas9/λ Red recombineering system has proven highly effective in multiple bacterial species for scarless genome editing (Ferreira et al. 2024; Jiang et al. 2015). Nevertheless, whether the λ Red system can be functionally integrated with CRISPR/Cas9 to establish a robust and efficient genome editing platform in *A. tumefaciens* , a species with inherently low homologous recombination capacity, remains unexplored.

Here, we developed and optimized a λ Red recombinase‐assisted CRISPR/Cas9 genome editing system in *A. tumefaciens* , allowing rapid, precise and scarless introduction of diverse mutation types into both the chromosome and Ti plasmids across different *A. tumefaciens* strains, with minimal off‐target effects. By incorporating *SacB*‐mediated plasmid curing, we streamlined strain generation without leaving behind genome editing vectors. Leveraging this platform, we generated an antibiotic‐sensitive strain and a host immunity‐evading strain, which together provide powerful tools for improving plant transformation efficiency and advancing synthetic biology applications in plants.

## 2 Results

### 2.1 The Intrinsic Homologous Recombination Capacity of *A tumefaciens* Is Insufficient to Repair Lethal DSB

A previous study demonstrated that constitutive expression of the CRISPR‐Cas9 system from a binary vector with pVS1 origin of replication (ORI) results in strong DNA cleavage activity in *A. tumefaciens* (Rodrigues et al. 2021). However, it was recognized that the highly constitutive Cas9 expression from vectors with a high copy number is toxic to many bacteria, affecting cell viability or growth (Zhao et al. 2020). To test whether the copy number of Cas9‐expression vectors influences the growth of *A. tumefaciens* , we constructed three CRISPR‐Cas9 vectors based on the replication origins pRi‐*repABC*, pVS1 and pBBR1 (Figure S1a). The pRi‐*repABC* ORI, derived from the *Agrobacterium rhizogenes* plasmid pRiA4b, replicates as a single copy per cell, whereas the broad‐host‐range ORIs pVS1 and BBR1 replicates as 9.5 and 52 copies per cell, respectively (Szarzanowicz et al. 2025; Vaghchhipawala et al. 2018). The regulatory elements driving Cas9 and sgRNA expression were identical to those previously reported (Rodrigues et al. 2021). The *Streptococcus pyogenes* Cas9‐coding sequence was under the control of a constitutive promoter *P*_*AGRO1*_, which was commonly used to drive antibiotic‐resistance genes in standard binary vectors. The expression of sgRNA was driven by a synthetic constitutive *J23119* promoter (Figure S1a). We observed that cell growth of *A. tumefaciens* was severely inhibited when Cas9 was expressed from the pBBR1‐Cas9‐SG vector harbouring pBBR1 ORI (Figure S1a). Given the cytotoxicity associated with of high‐copy‐number Cas9 expression, we next examined whether the CRISPR‐Cas9 system expressed from a low‐copy‐number vector was sufficient to cleave the genomic DNA in *A. tumefaciens* . The empty plasmid pRi‐Cas9‐SG and the *thymidylate synthase* gene (*thyA*)‐targeting plasmid pRi‐Cas‐SG‐*thyA* were transformed into *A. tumefaciens* strain EHA105. Transformation with the empty plasmid yielded a lawn of colonies, whereas no colonies were obtained with the *thyA*‐targeting plasmid (Figure S1b). This result indicates that Cas9 expressed from a single‐copy vector can cleave the genomic DNA of *A. tumefaciens* with high efficiency.

Although Cas9‐mediated chromosome cleavage is lethal to many bacteria, several reports have shown that bacteria can repair the DSBs generated by Cas9 via endogenous homologous recombination (HR) pathways if a repair template is provided (Collias et al. 2023). To evaluate the HR‐mediated DSB repair capacity of *A. tumefaciens* , we designed a two‐plasmid system in which the Cas9‐expression vector pRi‐Cas9 and the donor vector pBBR1‐SG‐Donor (carrying sgRNA and repair template) were co‐transformed into the *A. tumefaciens* EHA105 cells (Figure S1c). Although dozens of colonies appeared on the selection plate 4 days after transformation (Figure S1d), PCR screening showed that none of these colonies represented the desired *thyA* deletion mutants (Figure S1e). These findings suggest that the intrinsic HR capacity of *A. tumefaciens* EHA105 strain is insufficient to directly repair the lethal DSB created by CRISPR‐Cas9.

### 2.2 Establishment of a Lambada Red Recombinase‐Assisted CRISPR‐Cas9 System in *A. tumefaciens* EHA105 Strain

Bacteriophage‐derived recombinase systems, such as Redαβγ system from λ Red phage and RecE/T system from Rac prophage, can promote site‐specific homologous recombination in bacteria (Li, Li, et al. 2023). These systems have been successfully integrated with the CRISPR‐Cas9 system to achieve precise genome editing in several Gram‐negative bacteria (Ferreira et al. 2024; Jiang et al. 2015). Moreover, the λ Red system had been applied for recombineering of the *A. tumefaciens* genome, although the recombination efficiency was much lower than that in *E. coli* (Hu et al. 2014). Based on these findings, we hypothesized that introducing the exogenous λ Red recombination system could increase the HR capacity of *A. tumefaciens*. To this end, we constructed another two‐plasmid system consisting of pRi‐Cas9‐Red‐SacB and pBBR1‐SG‐Donor‐SacB (Figure 1a). In the pRi‐Cas9‐Red‐SacB plasmid, which harbours the pRi‐*repABC* ORI, the Cas9 nuclease is constitutively expressed from the *P*_*AGRO1*_ promoter, while expression of the λ Red operon is under the control of an anhydrotetracycline (aTc) inducible *P*_*tet*_ promoter. The pBBR1‐SG‐Donor‐SacB plasmid, carrying the pBBR1 ORI, contains a sgRNA expression cassette and a repair template flanked by homology arms (HA) (Figure 1a). Both plasmids harbour the *SacB* counter‐selection marker to facilitate plasmid curing after genome editing.

**FIGURE 1:**
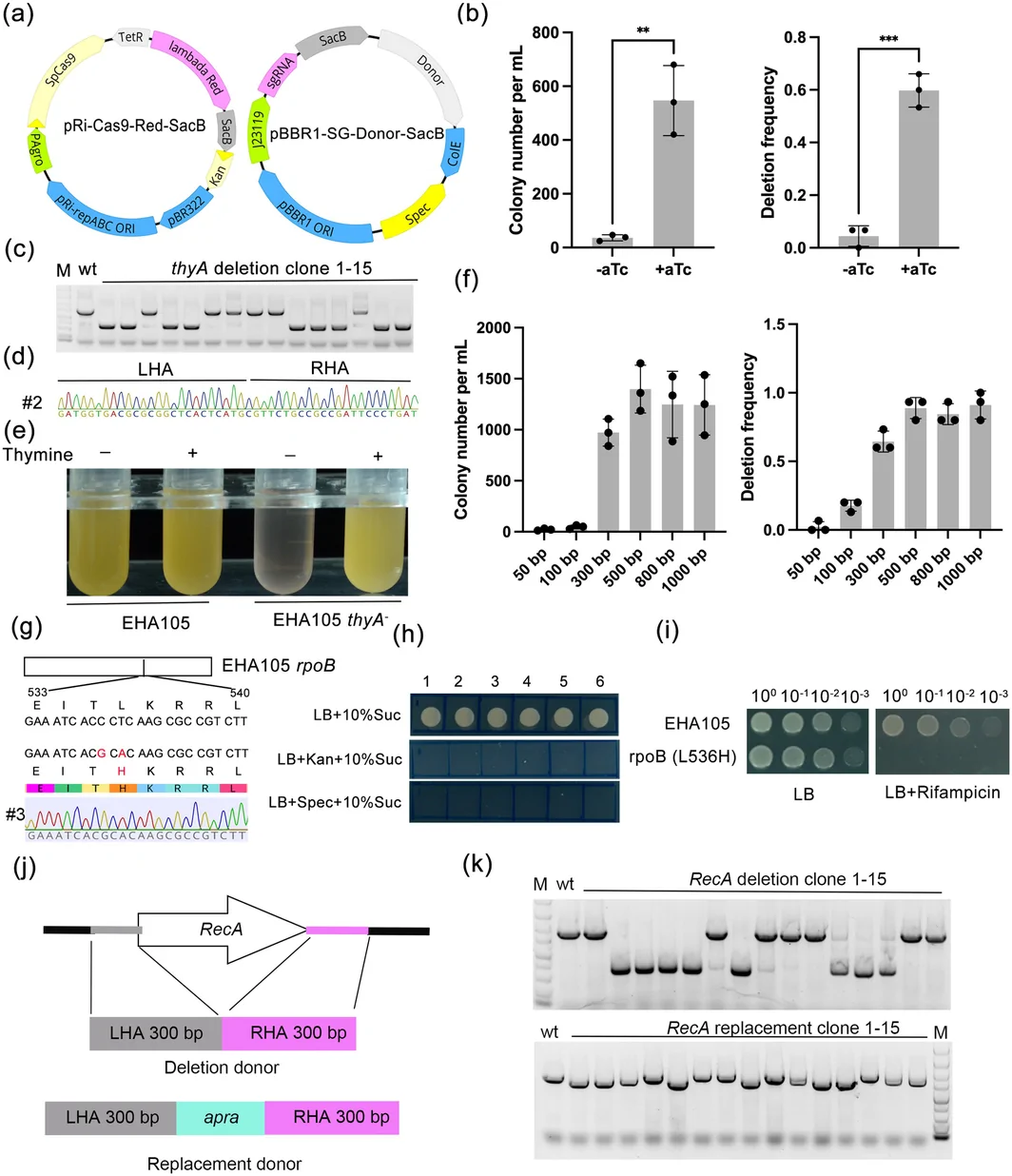
Establishment of a lambada Red recombinase‐assisted CRISPR‐Cas9 system in *A. tumefaciens* strain EHA105. (a) Schematic illustration of the two‐plasmid system for lambada Red recombinase‐assisted CRISPR‐Cas9 editing in *A. tumefaciens* . pRi‐Cas9‐Red‐SacB and pBBR1‐SG‐Donor‐SacB harbour the pRi‐*repABC* and pBBR1 origins of replication, respectively, enabling stable replication in *A. tumefaciens* . (b) Number of colonies and *thyA* deletion efficiency using the lambada Red recombinase‐assisted CRISPR‐Cas9 system with or without aTc induction of lambada Red operon expression. **p < 0.01 and ***p < 0.001 (Student's *t*‐test). (c) Successful deletion of *thyA* gene by the lambada Red recombinase‐assisted CRISPR‐Cas9 system, confirmed by PCR screening. (d) Sanger sequencing verified the precision of *thyA* deletion. Chromatograms from a representative edited colony (#2) was shown. (e) Thymidine‐dependent growth of auxotrophic *thyA* mutant strain in liquid LB medium supplemented with or without 100 mg/L of thymidine. (f) Colony yield and editing frequency for *thyA* deletion using donor templates with varying homologous arm lengths. Error bars represent SD from three independent experiments. (g) Schematic of base substitutions introduced at the *rpoB* gene in EHA105 strain. A L536H mutation was introduced. Sanger sequencing chromatograms at *rpoB* site from an edited colony (#3) was shown. (h) Plasmids curing via *SacB*‐mediated counter‐selection on 10% sucrose medium following *rpoB* editing. (i) Rifampicin sensitivity assay of the *rpoB* edited strain. The EHA105 serves as a wild‐type control. (j) Schematic view of donor designs for deletion or replacement of the *RecA* gene. (k) Verification of *RecA* deletion or replacement by PCR screening. In panels (c) and (k), the ‘wt’ lane corresponds to PCR amplification from wild‐type EHA105.

The pRi‐Cas9‐Red‐SacB plasmid was first transformed into the *A. tumefaciens* EHA105. A single colony was then used to prepare electrocompetent cells, either in the presence or absence of aTc inducer. These cells were subsequently electroporated with pBBR1‐SG‐Donor‐SacB plasmid, which was designed to target the *thyA* locus for deletion using a repair template with 300‐bp homology arms. The number of resulting colonies and the *thyA* deletion frequency were both significantly higher under aTc induction compared to the non‐induced condition (Figure 1b). We attribute the low editing rate under the non‐induced condition to potential leaky expression of λ Red operon. Sanger sequencing of colonies showing small PCR amplicons confirmed the desired deletions (Figure 1c,d). As expected, the *thyA* deletion rendered the *A. tumefaciens* strain auxotrophic for thymidine (Figure 1e).

Given that the recombination efficiency of λ Red system is highly dependent on the length of the HA in the repair template, we therefore evaluated the optimal HA length for efficient editing in *A. tumefaciens* . Donor templates with varying HA lengths were cloned into the *thyA*‐targeting pBBR1‐SG plasmid. Compared to the 300‐bp HA donor, constructs with 50‐bp or 100‐bp HAs produced significantly fewer colonies and exhibited lower editing efficiencies (Figure 1f). Conversely, increasing the HA length to 500‐bp, 800‐bp, or 1000‐bp only slightly improved the colony yield and the editing efficiency, suggesting that the 300‐bp HA donor was sufficient for robust and reliable editing (Figure 1f). Consequently, all subsequent experiments employed donors with either 300‐bp or 500‐bp HAs (Table 1). Using 300‐bp HA donors, we applied this two‐plasmid system to delete five additional genes, including *RecA*, *AmpC*, *MutS*, *MutL* and *Xse*, achieving deletion frequencies ranging from 26.7% to 86.7% (Figure 1j,k and Figure S2a).

**TABLE 1:** Summary of the mutation efficiency of different target sites in *Agrobacterium* strain EHA105, GV3101 and LBA4404.

Host	Target gene	Position	Vector	Length of donor arm (bp)	Mutation type^a^	Editing rate
EHA105	*thyA*	genome	pRi‐Cas9‐Red‐SacB	300 300	Deletion	60%
EHA105	*RecA*	genome	pRi‐Cas9‐Red‐SacB	300 300	Deletion	53.3%
EHA105	*RecA*	genome	pRi‐Cas9‐Red‐SacB	300 300	Replacement	60%
EHA105	*rpoB*	genome	pRi‐Cas9‐Red‐SacB	500 500	Substitution	100%
EHA105	*AmpC*	genome	pRi‐Cas9‐Red‐SacB	500 500	Deletion	60%
EHA105	*MutS*	genome	pRi‐Cas9‐Red‐SacB	300 300	Deletion	86.7%
EHA105	*MutL*	genome	pRi‐Cas9‐Red‐SacB	301 300	Deletion	26.7%
EHA105	*SSB*	genome	pRi‐Cas9‐Red‐SacB	300 303	Substitution	100%
EHA105	*Xse*	genome	pRi‐Cas9‐Red‐SacB	301 266	Deletion	26.7%
EHA105^b^	*thyA*	genome	pRi‐Cas9‐Red‐SacB‐lacIsg	300 300	Deletion	93.3%
EHA105^b^	*TetR*	genome	pRi‐Cas9‐Red‐SacB‐lacIsg	500 500	Deletion	80%
GV3101	*thyA*	genome	pRi‐Cas9‐Red‐SacB‐lacIsg	300 300	Deletion	73.3%
GV3101	*RecA*	genome	pRi‐Cas9‐Red‐SacB‐lacIsg	300 300	Deletion	60%
GV3101	*Xse*	genome	pRi‐Cas9‐Red‐SacB‐lacIsg	301 266	Deletion	40%
GV3101	*AmpC*	genome	pRi‐Cas9‐Red‐SacB‐lacIsg	500 500	Deletion	80%
GV3101	*rpoB*	genome	pRi‐Cas9‐Red‐SacB‐lacIsg	500 500	Substitution	100%
GV3101	*strB*	genome	pRi‐Cas9‐Red‐SacB‐lacIsg	500 501	Deletion	80%
GV3101^b^	*thyA*	genome	pRi‐Cas9‐Red‐SacB‐lacIsg	300 300	Deletion	46.6%
GV3101^b^	*RecA*	genome	pRi‐Cas9‐Red‐SacB‐lacIsg	300 300	Deletion	46.6%
EHA105	T‐DNA left border	pTiBo542	pRi‐Cas9‐Red‐SacB‐lacIsg	312 300	Replacement	100%
GV3101	*VirD2*	pMP90	pRi‐Cas9‐Red‐SacB‐lacIsg	303 300	Insertion	93.3%
GV3101	*VirD2*	pMP90	pRi‐Cas9‐Red‐SacB‐lacIsg	300 305	15 bp deletion	100%
EHA105	*cat*	genome	pRi‐Cas9‐Red‐SacB‐lacIsg	500 503	Deletion	100%
EHA105	*MltB3*	genome	pRi‐Cas9‐Red‐SacB‐lacIsg	500 500	Deletion	100%
EHA105	*strB*	genome	pRi‐Cas9‐Red‐SacB‐lacIsg	500 501	Deletion	86.7%
EHA105	*TetR*	genome	pRi‐Cas9‐Red‐SacB‐lacIsg	500 500	Deletion	93.3%
EHA105	*tufA*	genome	pRi‐Cas9‐Red‐SacB‐lacIsg	328 280	Substitution	93.3%
EHA105	*tufB*	genome	pRi‐Cas9‐Red‐SacB‐lacIsg	332 290	Substitution	93.3%
LBA4404	*RecA*	genome	pRi‐Cas9‐Red‐SacB‐lacIsg	500 500	Deletion	73.3%
LBA4404	*thyA*	genome	pRi‐Cas9‐Red‐SacB‐lacIsg	500 500	Deletion	80%

^a^ Substitution means single or multiple base conversions, whereas replacement means large fragment substitutions.

^b^ Second round of genome editing.

The λ Red system can utilize linear single‐strand or double‐stranded DNA as the repair template for recombination (Datsenko and Wanner 2000). We therefore assessed the editing efficiency of our system using linear double‐stranded DNA donors. We co‐transformed the linear PCR‐amplified donors and the pBBR1‐SG‐SacB plasmid (Figure S3a) into the competent cells harbouring the pRi‐Cas9‐Red‐SacB plasmid. For the *thyA* locus edited with a linear 300‐bp HA donor and the *AmpC* locus edited with a linear 500‐bp HA donor, the editing efficiency was only 6.7% for each (Figure S3b–e), substantially lower than those achieved with plasmid‐borne donors.

To extend the utility of the λ Red‐assisted CRISPR/Cas9 system beyond gene deletions, we tested its capacity to introduce point mutations. We designed an sgRNA and a donor DNA to repair the rifampicin‐resistance *rpoB* gene in strain EHA105. Sanger sequencing of fifteen clones confirmed a 100% editing frequency at this site (Figure 1g and Table 1). To cure the pRi‐Cas9‐Red‐SacB and pBBR1‐SG‐Donor‐SacB plasmids after genome editing, a single colony containing the desired L536H mutation was selected to inoculate into 10% sucrose‐containing LB medium for 48 h, and then a fraction of the cells was streaked onto a 10% sucrose‐containing LB agar plate and incubated at 28°C. Six individual colonies were randomly streaked onto 10% sucrose‐containing LB agar plates supplemented with either spectinomycin, kanamycin, or no antibiotic. None of the colonies grew on the antibiotic plates, confirming successful curing of both the pRi‐Cas9‐Red‐SacB and pBBR1‐SG‐Donor‐SacB plasmids (Figure 1h). As anticipated, the EHA105 *rpoB* (L536H) strain regained sensitivity to rifampicin (Figure 1i). We also introduced two base substitutions into the *SSB* gene to create an M181F mutation, again achieving an editing rate of 100% (Figure S2b). Finally, to assess the system's capacity for large fragment replacement, we designed a donor to replace the *RecA* gene with an apramycin resistance gene, resulting in an editing frequency of 60% (Figure 1j,k). Collectively, the above results demonstrate that the λ Red‐assisted CRISPR/Cas9 system can introduce versatile mutation types in *A. tumefaciens* with high efficiency.

### 2.3 A Modified Two‐Plasmid System for Sequential Gene Editing

After successful editing, both pRi‐Cas9‐Red‐SacB and pBBR1‐SG‐Donor‐SacB plasmids were cured, which required re‐introducing the pRi‐Cas9‐Red‐SacB plasmid for each subsequent editing round. To simplify the process of editing multiple genes, we constructed an improved plasmid pRi‐Cas9‐Red‐SacB‐lacIsg by integrating into the pRi‐Cas9‐Red‐SacB backbone an IPTG‐inducible sgRNA expression cassette targeting the *Rep* gene of the pBBR1 ORI (Figure 2a). This design allows for the inducible elimination of the pBBR1‐SG‐Donor‐SacB vector between editing cycles. Following the first editing cycle, the edited clone was inoculated into liquid medium supplemented with IPTG to induce *Rep*‐targeting sgRNA expression, thereby eliminating the pBBR1‐SG‐Donor‐SacB vector. The overnight culture was then spread on a kanamycin‐containing LB plate. Once the loss of spectinomycin resistance was confirmed, the clone was used to prepare competent cells for the next round of editing (Figure 2b). We first assessed whether incorporating the *Rep*‐targeting sgRNA would compromise the transformation and editing efficiency. The colony numbers and editing frequencies at the *thyA* and *AmpC* loci were comparable between strains carrying the original pRi‐Cas9‐Red‐SacB plasmid and the modified pRi‐Cas9‐Red‐SacB‐LacIsg plasmid (Figure 2c–e). To evaluate the utility of the pRi‐Cas9‐Red‐SacB‐LacIsg plasmid for continual gene editing, we conducted sequential gene deletions. We first deleted the *RecA* gene and then eliminated the pBBR1‐SG‐Donor‐SacB vector in edited clones via IPTG induction. Subsequently, we deleted the *thyA* and *tetR* genes in the *RecA* mutant background, achieving mutation efficiencies of 93.7% and 80%, respectively (Figure 2f). These results indicate that integrating an inducible, *Rep*‐targeting sgRNA cassette into the pRi‐Cas9‐Red‐SacB plasmid enables iterative editing cycles without sacrificing transformation and editing efficiency. Furthermore, highly efficient editing in the *RecA* mutant background demonstrates that the repair of CRISPR/Cas9‐generated DSB relies on the exogenous λ Red system and is independent of the endogenous *RecA*‐mediated homologous recombination pathway.

**FIGURE 2:**
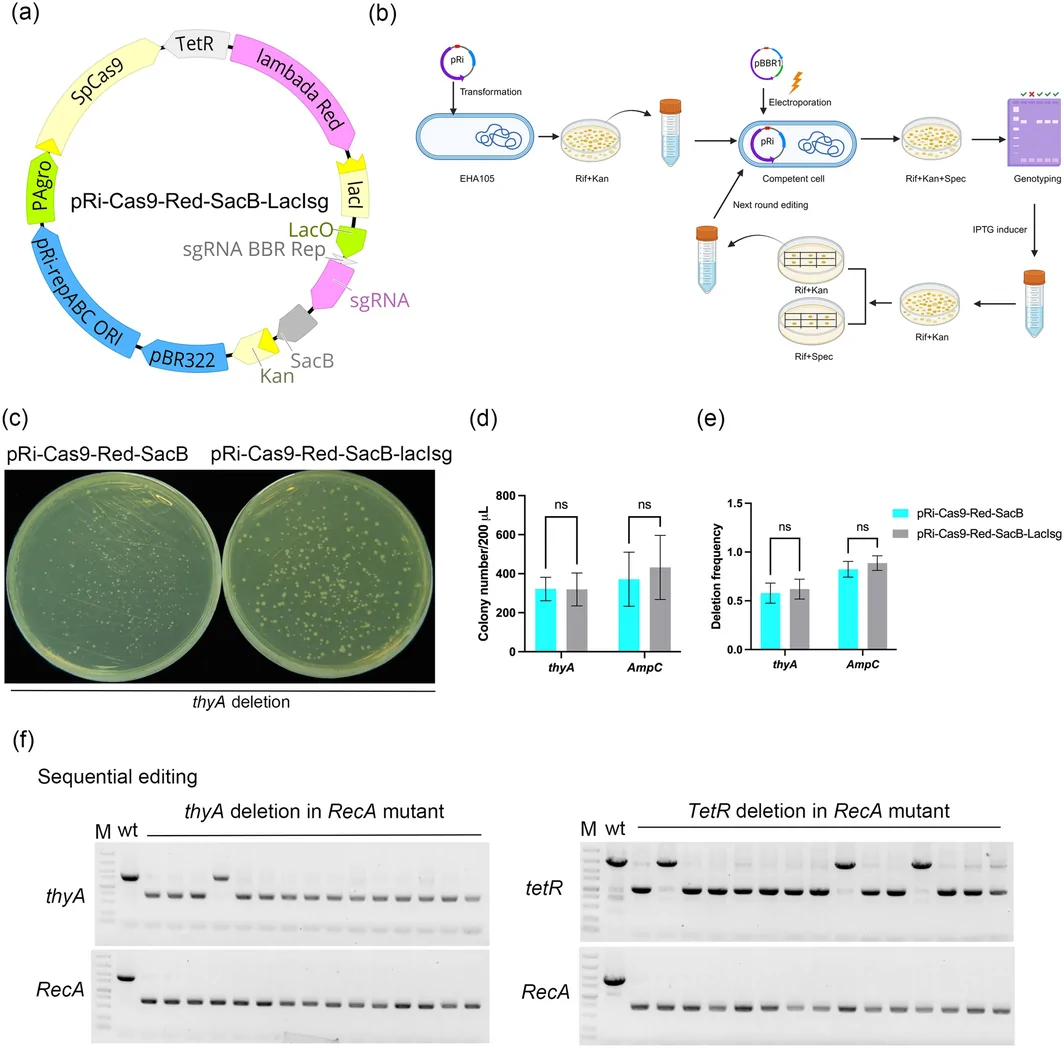
A modified two‐plasmid system for sequential gene editing. (a) Schematic of the pRi‐Cas9‐Red‐SacB‐lacIsg plasmid. An IPTG‐inducible sgRNA expression cassette targeting the *Rep* gene of the pBBR1 origin of replication was inserted into the pRi‐Cas9‐Red‐SacB to generate pRi‐Cas9‐Red‐SacB‐lacIsg plasmid. (b) Workflow for sequential gene editing using the modified two‐plasmid system. Schematics were created using BioRender. (c) Colony formation on selective plates using pRi‐Cas9‐Red‐SacB and pRi‐Cas9‐Red‐SacB‐LacIsg plasmids for *thyA* editing. One‐fifth of each transformation mixture was plated onto the selective plates. (d, e) Comparison of colony number (d) and editing frequency (e) at *thyA* and *AmpC* loci when use pRi‐Cas9‐Red‐SacB and pRi‐Cas9‐Red‐SacB‐LacIsg plasmids for editing. ns, not significant (Student's *t*‐test). (f) Sequential editing of *thyA* and *tetR* in a *RecA* mutant background. *thyA* and *tetR* deletion was detected by PCR screening. The ‘wt’ lane represents PCR amplification from wild‐type EHA105.

### 2.4 Engineering the *A. tumefaciens* GV3101 Strain

The efficiency of CRISPR‐Cas9‐based genome editing can vary among bacterial strains of the same species (Li et al. 2021). To evaluate the broader applicability of our λ Red‐assisted CRISPR/Cas9 system, we then tested its performance in another commonly used laboratory strain GV3101, which shares the C58 genome background with EHA105 strain but harbours a different Ti plasmid, pMP90. The GV3101 genome contained an R523Q mutation in the *rpoB* gene, which confers rifampicin resistance (Figure 3a). We therefore designed an sgRNA and a repair donor to revert this mutation to the wild‐type sequence. Using the two‐vector system comprising pRi‐Cas9‐Red‐SacB‐lacIsg and pBBR1‐SG‐Donor‐SacB, we achieved an editing efficiency of 100% at this site and the edited clone became sensitive to rifampicin (Figure 3b). To further assess the versatility of the system in GV3101, we targeted four additional genes (*thyA, RecA*, *Xse* and *AmpC*) for deletion, achieving editing efficiencies ranging from 40% to 80% (Figure 3c). To test the feasibility of sequential editing in this strain, we first deleted the *strB* gene and then removed the pBBR1‐SG‐Donor‐SacB vector in edited clones via IPTG induction. Subsequently, we deleted the *RecA* and *thyA* individually in the *strB‐*deficient background, achieving an editing efficiency of 40% for each target (Figure 3d).

**FIGURE 3:**
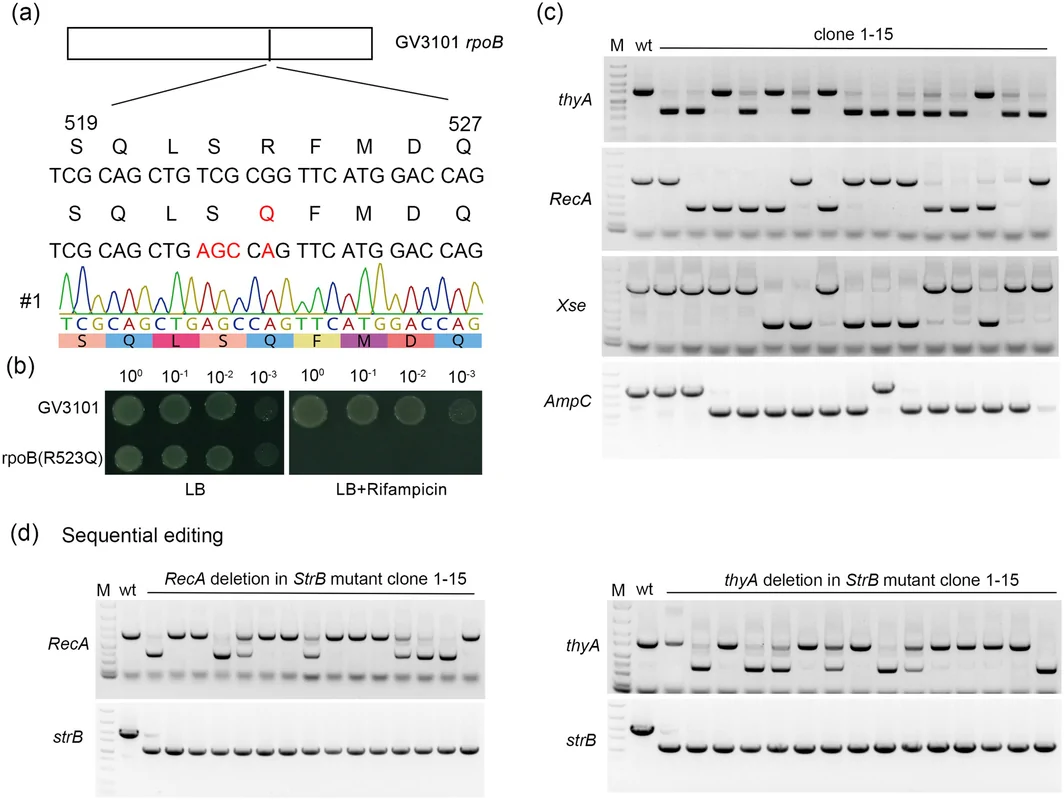
Engineering the *A. tumefaciens* GV3101 strain using the modified two‐plasmid system. (a) Schematic of the R523Q mutation introduced in the *rpoB* gene of the GV3101 strain. Sanger sequencing chromatograms from an edited colony (#1) was shown. (b) Rifampicin sensitivity assay of the *rpoB* edited strain. The GV3101 serves as a wild‐type control. (c) Deletion of the *thyA*, *RecA*, *Xse*, *AmpC* genes in GV3101 by the modified two‐plasmid system. The deletion events were detected by PCR screening. (d) Sequential editing of *RecA* and *thyA* in a *strB* mutant background. *RecA* and *thyA* deletion was verified by PCR screening. The ‘wt’ lane represents PCR amplification from wild‐type GV3101.

Because EHA105 and GV3101 both have a C58 chromosomal background, we also tested the λ Red recombinase‐assisted CRISPR/Cas9 system in *A. tumefaciens* LBA4404, another widely used laboratory strain with an Ach5 background. Using the modified two‐plasmid system, we first targeted *RecA* for deletion. Without aTc induction of λ Red operon expression, no *RecA* deletion events were detected among fifteen randomly selected colonies. By contrast, aTc induction results in a deletion frequency of 73.3% (Figure S4a). We then targeted *thyA* for deletion, achieving an editing efficiency of 80% (Figure S4b and Table 1). Sanger sequencing of edited colonies confirmed the precise deletion of the *thyA* gene (Figure S4c), and the resulting LBA4404 *thyA* ^*−*^ mutant exhibited thymine‐dependent growth (Figure S4d). To evaluate the sequential editing in LBA4404, we cured the pBBR1‐SG‐Donor‐SacB vector after *RecA* deletion and subsequently deleted the *thyA* gene in the *RecA* mutant background, achieving an editing frequency of 73.3% (Figure S4e). This result further confirms that the λ Red recombinase‐assisted CRISPR/Cas9 system operates independently of the endogenous *RecA*‐mediated homologous recombination pathway for DSB repair. Taken together, these results demonstrate that the λ Red‐assisted CRISPR/Cas9 system enables efficient genome editing across different *A. tumefaciens* strains.

### 2.5 Engineering Ti Plasmids in Different *A. tumefaciens* Strains

The disarmed Ti plasmid is essential for *Agrobacterium*‐mediated plant transformation. We thus tested whether the λ Red recombinase‐assisted CRISPR/Cas9 system could edit Ti plasmids. Previous whole‐genome sequencing showed that the disarmed pEHA105 Ti plasmid retains the left border sequence of left T‐DNA in the hypervirulent pTiBo542 plasmid (Rodrigues et al. 2021). We therefore designed an sgRNA and a donor DNA to replace this left border and a downstream 1.74 kb sequence with a gentamycin resistance cassette (Figure 4a). PCR screening of the resulting colonies indicated that the editing frequency was 100% (Figure 4a). Next, we examined whether this replacement affects plant transformation efficiency. We used both wild‐type EHA105 strain and the engineered EHA105‐GenR strain to deliver three prime editor vectors into rice. Although absolute transformation efficiency showed large variations, no significant difference was observed between the two strains (Figure 4b). We then edited the *VirD2* gene in the pMP90 Ti plasmid of strain GV3101. The omega domain deletion of VirD2 impairs T‐DNA integration into the plant genome, whereas insertion of a 3xHA tag along with a GFP_11_ fragment enables monitoring the trafficking of VirD2 protein in plant cells during the *Agrobacterium*‐mediated transformation by using the split‐GFP assay (Mysore et al. 1998; Roushan et al. 2018). Using the λ Red‐assisted CRISPR/Cas9 system, we successfully introduced these two distinct mutations into *VirD2*, achieving editing efficiencies of 93.3% and 100%, respectively (Figure 4c and Table 1). In summary, these results demonstrate that our λ Red‐assisted CRISPR/Cas9 system can efficiently edit the Ti plasmids in different *A. tumefaciens* strains.

**FIGURE 4:**
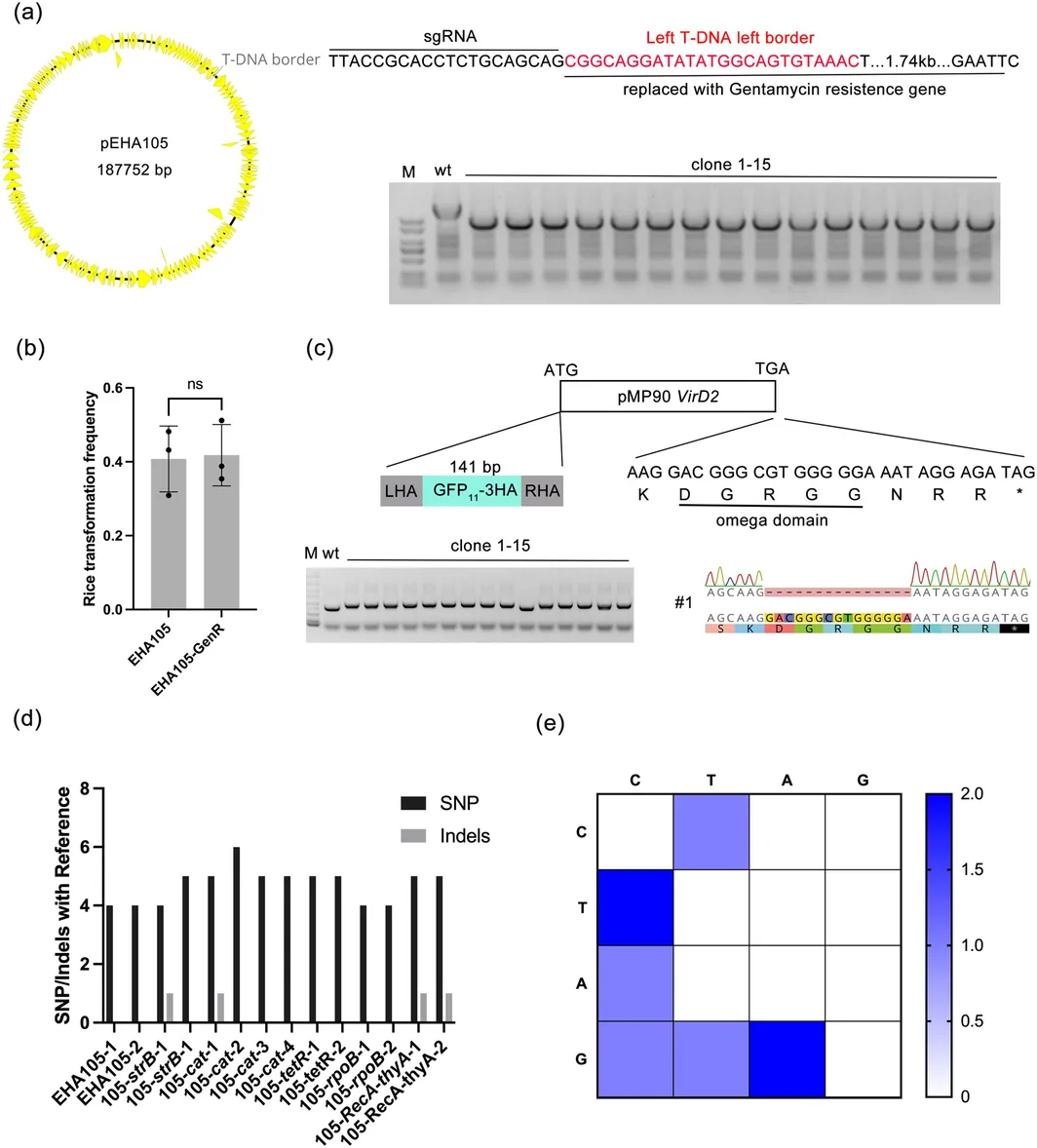
Engineering Ti plasmids and genome‐wide off target analysis. (a) Elimination of a T‐DNA left border sequence in pEHA105 Ti plasmid. The T‐DNA left border sequence and a downstream 1.74 kb sequence were replaced with a gentamycin resistance cassette. Successful replacement was confirmed by PCR screening. (b) Comparison of plant transformation efficiency between the wild‐type EHA105 strain and the engineered EHA105‐GenR strain. ns, not significant (Student's *t*‐test). (c) Engineering the *VirD2* gene in the pMP90 Ti plasmid by either inserting a 3xHA tag along with a GFP_11_ fragment at the N‐terminus or deleting the omega domain at the C‐terminus. The GFP_11_‐3HA insertion was verified by PCR screening; the omega domain deletion was confirmed by Sanger sequencing. (d) Total number of single‐nucleotide variants (SNVs) and insertions/deletions (indels) detected in the genome of EHA105 lab strains and edited strains relative to the reference genome. (e) Preference profiles of SNV types in edited strains after filtering out background mutations native to the EHA105 lab strain.

### 2.6 Whole‐Genome Sequencing Reveals Low Off‐Target Editing

To evaluate the potential off‐target editing by our λ Red recombinase‐assisted CRISPR/Cas9 system, we performed whole‐genome sequencing on two wild type EHA105 clones used in our lab and twelve edited clones in the EHA105 background that had been cured of the editing plasmids. SNP analysis revealed that the EHA105 strain used in our lab carried four SNPs relative to the published reference genome (Figure 4d). After excluding these background SNPs, no off‐target editing was observed in the two *rpoB*‐edited clones. The other edited clones contained only one or two SNPs compared to wild‐type EHA105 control. Notably, four edited clones each had one indel in their genome (Figure 4d). The sequences of all mutation sites were divergent from the sgRNA spacers, suggesting that the mutations were sgRNA‐independent. Moreover, no base substitution bias was observed among the SNPs (Figure 4e). Together, these findings demonstrate that our system is highly specific, with minimal off‐target activity.

### 2.7 Engineering the Antibiotic‐Sensitive *A. tumefaciens* Strain

Both the EHA105 and GV3101 strains are intrinsically resistant to several antibiotics, including rifampicin, β‐lactams, chloramphenicol and streptomycin (Figueroa‐Cuilan et al. 2022; Howarth et al. 2023; Tennigkeit and Matzura 1991). Additionally, although normally sensitive to tetracycline, these strains readily generate spontaneous tetracycline‐resistant mutants at a high frequency due to a latent resistance determinant in their genome (Luo and Farrand 1999). GV3101 also harbours a gentamycin resistance gene on the disarmed pMP90 Ti plasmid (De Saeger et al. 2021). The prevalence of these antibiotic resistance genes complicates various applications. For instance, during plant transformation, high concentrations of β‐lactam antibiotics are often required to control *A. tumefaciens* overgrowth due to this resistance, which can adversely affect plant tissue growth and regeneration. Additionally, when transferring synthetic biology tools from other model bacteria into *A. tumefaciens* , the need to exchange antibiotic selection markers complicates the process.

The key intrinsic resistance determinants in *A. tumefaciens* strains include the *cat* gene, encoding chloramphenicol acetyltransferase, the *strB* gene, encoding a StrB family phosphotransferase involved in streptomycin inactivation, and the *AmpC* gene, encoding a putative inducible β‐lactamase (Figueroa‐Cuilan et al. 2022; Howarth et al. 2023; Tennigkeit and Matzura 1991). While deletion of *AmpC* causes a viability defect on solid medium, mutation of *MltB3*, which encodes a cell wall remodelling enzyme essential for AmpC‐dependent β‐lactamase activity, confers sensitivity to ampicillin and carbenicillin without affecting cell viability (Figueroa‐Cuilan et al. 2022). Therefore, we targeted *MltB3* to abrogate β‐lactam resistance. Using our modified two‐plasmid editing system (Figure 2a), we sequentially deleted the *tetR*, *strB*, *cat* and *MltB3* genes in strain EHA105 (Figure S5). After curing the editing plasmids, we tested the antibiotic sensitivity of single, double, triple and quadruple mutants. As expected, the quadruple mutant (designated EHA105‐S) became sensitive to tetracycline, streptomycin, chloramphenicol and ampicillin, in contrast to the wild‐type strain (Figure 5a). To determine if the gene deletions impaired bacterial fitness, we compared the growth of EHA105 and EHA105‐S in liquid culture by monitoring OD_600_. The growth curve of EHA105‐S was indistinguishable from that of the EHA105 (Figure 5b). We also assessed whether the mutations affected transformation efficiency using *Agrobacterium*‐mediated transient expression of RUBY and GFP reporter genes in *Nicotiana benthamiana* leaves. No significant difference in signal intensity was observed between leaves infiltrated with EHA105 and those infiltrated with EHA105‐S (Figure 5c–f), indicating that the loss of antibiotic resistance genes did not compromise transient transformation efficiency. In summary, our results demonstrate that deleting the *tetR*, *strB*, *cat* and *MltB3* genes does not adversely affect the growth or transformation efficiency of *A. tumefaciens strain EHA105*.

**FIGURE 5:**
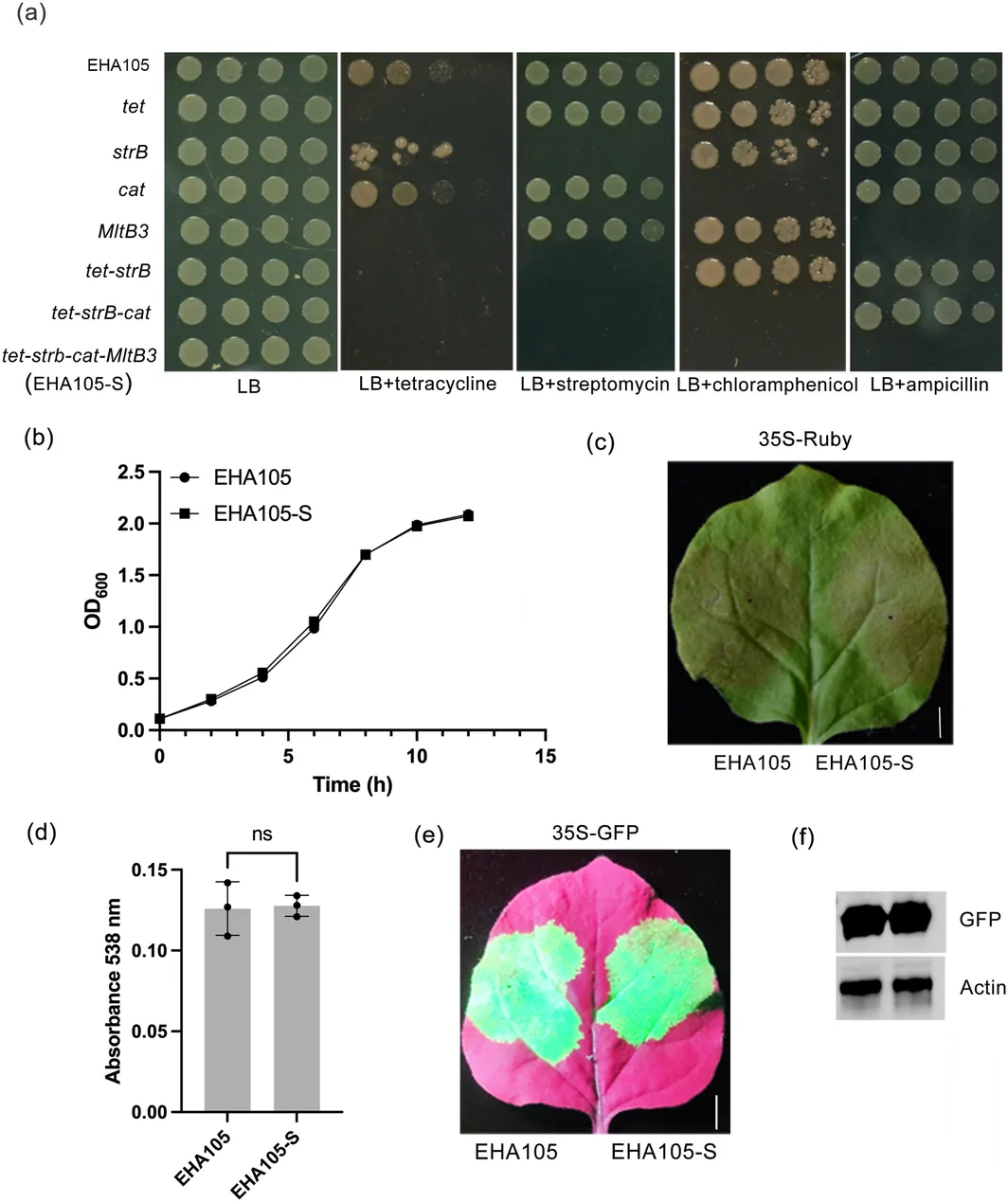
Engineering the antibiotic‐sensitive *A. tumefaciens* strain. (a) Antibiotic susceptibility assay of edited strains. Serial dilutions of exponential‐phase cultures were spotted onto LB solid medium containing no antibiotic or varying concentrations of antibiotics. EHA105 serves as a wild‐type control. (b) Growth curves of EHA105 and EHA105‐S monitored at 600 nm over time using a spectrophotometer. (c) Transient expression of a *RUBY* reporter in the *N. benthamiana* leaves infiltrated with EHA105 or EHA105‐S strains. (d) Quantification of betalain concentration in (c) by light absorbance at 538 nm. ns: Not significant (Student's *t*‐test). (e) Transient expression of a *GFP* reporter in the *N. benthamiana* leaves infiltrated with EHA105 or EHA105‐S strains. (f) Detection of GFP expression in (e) by Western blot analysis. Scale bar in (c) and (e) is 0.5 cm.

### 2.8 Engineering *Agrobacterium* EF‐Tu Genes to Enhance Transient Gene Expression in *Arabidospsis*

During *Agrobacterium‐mediated* plant transformation, microbe‐associated molecular patterns (MAMPs) from *A. tumefaciens* can activate pattern‐triggered immunity (PTI) via host plant receptors, thereby limiting transformation efficiency. Among these MAMPs, the EF‐Tu (or its conserved epitope elf18) is perceived by EF‐Tu receptor (EFR) in the Brassicaceae plants. This EFR‐dependent immunity is a major barrier to efficient transformation in this family, which includes *Arabidopsis thaliana* (Zipfel et al. 2006). *Agrobacterium* C58 genome harbours two tandem EF‐Tu‐encoding genes, *tufA* and *tufB*, separated by a 19 177 bp sequence (Figure 6a). Both genes are essential and cannot be deleted. However, substituting the N‐terminal elf18 epitope (amino acids 1–18) of *Agrobacterium* EF‐Tu with the homologous sequence from *Pseudomonas syringae* pv *tomato* (*Pst*) DC3000 EF‐Tu has been shown to reduce PTI activation and enhance transient expression in *Arabidopsis* leaves (Liu et al. 2026; Yang et al. 2023). We therefore used our λ Red‐assisted CRISPR/Cas9 system to replace the elf18 epitope of *Agrobacterium tuf* genes with the corresponding elf18 sequence from *Pst* EF‐Tu (Figure 6b and Figure S6a–e), generating single *tufA* ^*DC3000 4‐15*^, *tufB* ^*DC3000 4‐15*^ and double *tufA* ^*DC3000 4‐15*^ *tufB* ^*DC3000 4‐15*^ substitution mutants in EHA105 background. Following plasmid curing, we monitored the growth of these strains alongside the wild‐type EHA105 in liquid culture. While the *tufA* ^*DC3000 4‐15*^ and *tufB* ^*DC3000 4‐15*^ strains grew comparably to EHA105, the *tufA* ^*DC3000 4‐15*^ *tufB* ^*DC3000 4‐15*^ strain exhibit a significant growth defect (Figure 6c). To assess their transformation capacity, we performed transient expression assays in 3‐day‐old *A. thaliana* seedlings using a GUS reporter. Strains with single *tuf* gene substitution showed significantly higher transient GUS activity than the wild type. The *tufA* ^*DC3000 4‐15*^ *tufB* ^*DC3000 4‐15*^ strain produced even greater GUS activity than the strains with single *tuf* gene substitution (Figure 6d,e). Consistent with *Nicotiana benthamiana* lacking an EFR ortholog, transient assays in *Nicotiana benthamiana* leaves revealed no significant differences in GUS staining intensity among any of the strains (Figure S6f).

**FIGURE 6:**
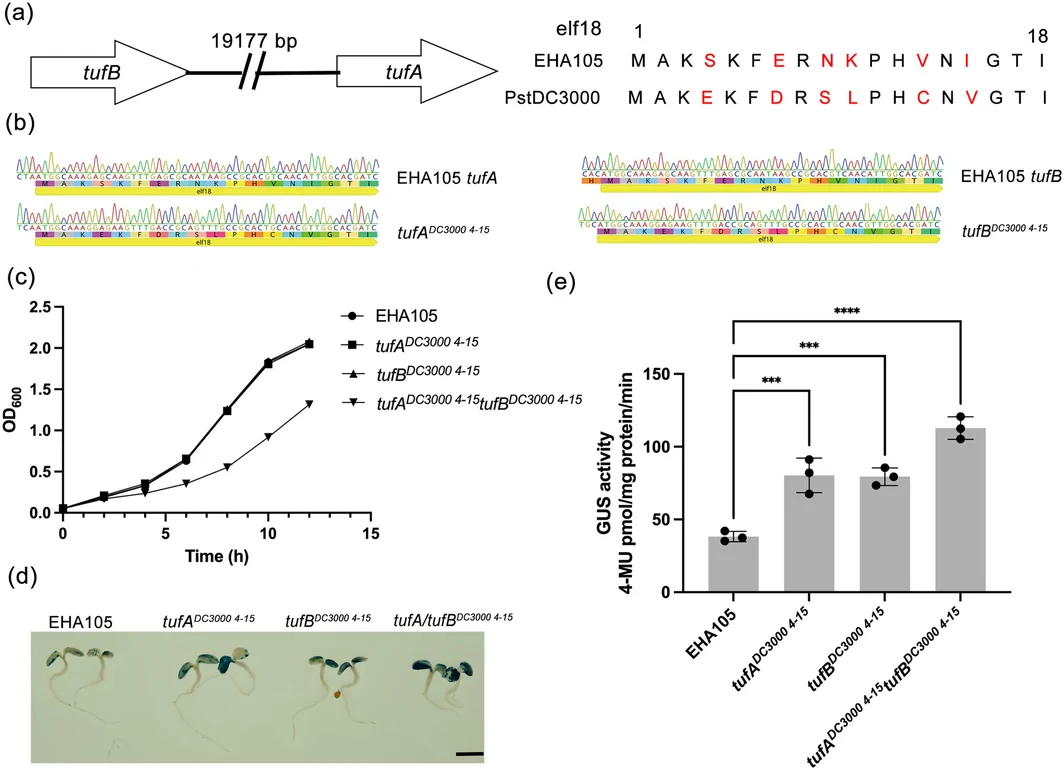
Engineering *Agrobacterium* EF‐Tu genes to enhance transient gene expression in *Arabidospsis*. (a) Left: Schematic illustration of *tufA* and *tufB* gene in the *A. tumefaciens* genome; Right: Alignment of the N‐terminus 18 amino acid sequences (elf18) of EF‐Tu genes between *A. tumefaciens* EHA105 and *Pseudomonas syringae* pv *tomato* (*Pst*) DC3000. The different residues are highlighted in red. (b) Sanger sequencing chromatograms confirmed the precise base substitutions in the elf18 epitope region of both *tufA* and *tufB* genes. (c) Growth curves of EHA105, *tufA* ^*DC3000 4‐15*^, *tufB* ^*DC3000 4‐15*^ and double *tufA* ^*DC3000 4‐15*^ *tufB* ^*DC3000 4‐15*^ mutant strains, monitored at 600 nm over time using a spectrophotometer. (d) Transient GUS expression efficiency in three‐day old *Arabidopsis* seedlings infiltrated with EHA105, *tufA* ^*DC3000 4‐15*^, *tufB* ^*DC3000 4‐15*^ and *tufA* ^*DC3000 4‐15*^ *tufB* ^*DC3000 4‐15*^ strains. Scale bar: 0.5 cm. (e) Quantification of GUS activity in (d) using the fluorometric substrate 4‐methylumbelliferyl‐β‐D‐glucuronide (MUG). ***p < 0.001 and ****p < 0.0001 (Student's *t*‐test).

## 3 Discussion

Genetic transformation was a bottleneck for plant genetic engineering and genome editing (Wang et al. 2025). *A. tumefaciens* is the most important and reliable vehicle for plant genetic transformation. Engineering *A. tumefaciens* strains to control bacterium overgrowth during infection, increase *Vir* gene expression or activity, or evade host immune response is valuable for enhancing plant transformation efficiency (Goralogia et al. 2025). However, traditional HR‐based genetic manipulation methods for engineering *A. tumefaciens* are time‐consuming and inefficient. The widely used laboratory strains have been used for more than 40 years without further improvements (De Saeger et al. 2021). In this study, by coupling the λ Red recombination system to the CRISPR/Cas9 genome cleavage system, we developed a highly efficient genome editing system in *A. tumefaciens* , allowing for efficient and precise gene deletion, insertion, base substitutions and large fragment replacement in different *A. tumefaciens* strains with minimal off‐target editing (Table 1).

*A. tumefaciens* exhibits intrinsic resistance to multiple antibiotics (Figure 5a). This resistance profile limits the choice of selective reagents during bacterial transformation, resulting in the common use of kanamycin and spectinomycin. In synthetic biology applications, which frequently require maintaining multiple plasmids with distinct selection markers, this reliance on specific antibiotics constrains the use of *A. tumefaciens* as a chassis. Furthermore, it hinders the exchange of synthetic biology tools between *A. tumefaciens* and other model bacteria, as the selection markers may need to be swapped to ensure compatibility. We used λ Red‐assisted CRISPR/Cas9 system to sequentially delete four antibiotic‐resistance loci in strain EHA105. The resulting strain EHA105‐S is sensitive to multiple antibiotics and retains wild‐type growth and transient transformation efficiency (Figure 5c–e). This engineered, antibiotic‐sensitive strain will serve as a valuable new host for future synthetic biology tools development in *A. tumefaciens* .

Suppressing the host immune response during *Agrobacterium‐mediated* plant transformation is an effective strategy for enhancing transformation efficiency (Raman et al. 2022). Although the flagellin and peptidoglycan MAMPs of *A. tumefaciens* can evade recognition by *Arabidopsis* PRRs, the EF‐Tu MAMP (its epitope elf18) is perceived by the *Arabidopsis* PRR EFR, triggering an immune response that reduces the transient transformation efficiency (Zipfel et al. 2006). In this study, we used sequential editing to engineer the two EF‐Tu genes in *Agrobacterium* to escape EFR perception. The resulting strain, carrying the *tufA* ^*DC3000 4‐15*^ *tufB* ^*DC3000 4‐15*^ mutations, improved transient transformation efficiency in *Arabidopsis* (Figure 6d,e). Since the EFR receptor is widespread in the Brassicaceae family, this engineered strain may benefit the transformation of crops in this family, including many important vegetables and oilseeds.

Recently, two CRISPR‐based genome editing tools, cytosine base editors (CBEs) and CRISPR RNA‐guided transposase system INTEGRATE, were developed in *A. tumefaciens* (Aliu et al. 2022; Rodrigues et al. 2021). However, both platforms possess certain drawbacks. CBEs are restricted to C‐T (or G‐A) point mutations, their targeting scope is constrained by PAM sequence, and the deaminase component can induce bystander mutations or genome‐wide sgRNA‐independent off‐target editing. The INTEGRATE system can efficiently generate DNA insertions and large DNA fragment deletions, yet the integration process can leave behind scar sequences. Furthermore, for the sequential insertional mutagenesis, there is a risk that a previously integrated cargo may be remobilized if the same INTEGRATE system is reused. Our λ Red‐assisted CRISPR/Cas9 system overcomes these drawbacks by enabling the precise, iterative, scar‐free introduction of all types of mutations with relaxed PAM sequence constraints. Moreover, whole‐genome sequencing of edited strains demonstrates that our editing system is highly specific (Figure 4b–e).

The *RecA* gene in bacteria facilitates DNA repair via homologous recombination. In *A. tumefaciens* , *RecA* mutation has been employed to stabilize the transformed binary vector, as exemplified by the widely used AGL1 strain (Lazo et al. 1991). However, this mutation simultaneously impedes conventional HR‐based genetic engineering, which relies on a functional *RecA* gene (Goralogia et al. 2025). Our sequential editing experiments demonstrate that the λ Red‐assisted CRISPR/Cas9 system achieves highly efficient gene editing even in the *RecA* mutant background (Figures 2f and 3d). This result indicates that the DSBs generated by CRISPR/Cas9 are mainly repaired by the introduced λ Red recombinase, rather than the endogenous *RecA* pathway. Consequently, our system will be valuable in the genome engineering of *RecA*‐deficient strains like AGL1.

The λ Red system can integrate the linear ssDNA or dsDNA donors into the genome via homologous recombination with high efficiency (Li, Li, et al. 2023). However, using our λ Red‐assisted CRISPR/Cas9 system, we observed that both colony numbers and editing efficiencies were substantially lower with linear PCR donors compared to plasmid‐borne donors (Figure S3). This reduction likely stems from lower transformation efficiency and/or rapid degradation of linear DNA donors in *A. tumefaciens* . While previous studies demonstrated that the λ Red system can recombine donors with HA as short as 50‐bp in many bacteria (Corts et al. 2022), our results show that plasmid‐borne donors require HAs of at least 100‐bp to achieve an acceptable editing rate in *A. tumefaciens* (Figure 1f). The precise molecular mechanism underlying the λ Red‐assisted CRISPR/Cas9 editing in *A. tumefaciens* remains to be elucidated.

CRISPR/Cas9‐mediated genomic DNA cleavage is lethal to bacteria and is therefore frequently used as a counter‐selection strategy to enrich correctly edited clones. However, in our system, we observed that some *A. tumefaciens* clones retained the wild type target sequences despite the presence of CRISPR/Cas9 targeting. This phenomenon is consistent with reports in other bacteria. These escapers likely arise from mutations or deletions in the plasmid‐encoded sgRNA or *Cas9* gene, which would preclude self‐targeting (Li, Sun, et al. 2023). The precise factors contributing to the emergence of these escaper clones remain to be investigated.

The λ Red system functions efficiently in *E. coli* and closely related species but exhibited suboptimal activity in more distantly related microbes such as *Agrobacterium* (Hu et al. 2014). Developing *Agrobacterium*‐specific recombineering systems by mining the homologous recombination machinery from *Agrobacterium* phages could therefore further increase the editing efficiency of our platform. A previous study using RecT as a query in PSI‐BLAST searches of publicly deposited *Agrobacterium* and *Rhizobium* genomic databases has identified candidate *Agrobacterium* phage‐encoded recombineering operons (Bian et al. 2022). The four tested recombineering operons exhibited various recombination efficiencies in different *Agrobacterium* strains (Bian et al. 2022). Coupling these recombinases with our CRISPR/Cas9 system may offer a path to achieve higher editing efficiency in *A. tumefaciens*.

In summary, we successfully developed a λ Red recombinase‐assisted CRISPR/Cas9 genome editing system for *A. tumefaciens* . This system enables precise introduction of versatile mutations into both the chromosome and Ti plasmid of different *A. tumefaciens* strains. Our platform can accelerate gene functional analyses in *A. tumefaciens* and holds great potential for developing optimized strains to improve plant transformation efficiency and facilitate broader synthetic biology applications in plants.

## 4 Materials and Methods

### 4.1 Bacterial Strains, Media and Culture Conditions

*E.coli* strains DH5𝛂 and DB3.1 were used as the cloning hosts for plasmid construction. *A. tumefaciens* strains GV3101, EHA105 and LBA4404 were purchased from the commercial company (WEIDI, Shanghai) and served as parental strains for genome editing. *E.coli* was cultured in the Luria Bertani (LB) medium (1% tryptone, 0.5% yeast extract, 1% NaCl) at 37°C, supplemented with appropriate antibiotics: 50 μg/mL kanamycin, 100 μg/mL spectinomycin and 50 μg/mL carbenicillin. *A. tumefaciens* strains were grown at 28°C in LB medium with following antibiotic concentrations: 50 μg/mL for kanamycin, 100 μg/mL for spectinomycin, 25 μg/mL for rifampicin, 25 μg/mL for chloramphenicol, 100 μg/mL for streptomycin, 50 μg/mL for ampicillin, 50 μg/mL for gentamicin and 10 μg/mL for tetracycline. For plasmid curing after genome editing, 10% (w/v) sucrose was added to LB medium. To sustain the growth of auxotrophic *thyA* mutant, 100 mg/L thymidine was supplemented into the LB medium.

### 4.2 Vector Construction

The constitutive promoter P_*AGRO1*_, driving SpCas9 expression in *A. tumefaciens* , was adapted from a previous study (Rodrigues et al. 2021). The sequence of P_*AGRO1*_ was synthesized by Qingke (Shanghai). The bacteria codon‐optimized SpCas9 sequence was amplified from SpCas9 nickase in pCRISPR‐PE‐bacteria plasmid (Addgene #172715) with the H840A mutation reversing back to wild type. To construct the SpCas9 expression cassette, the P_*AGRO1*_ promoter, SpCas9 coding sequence and T3 terminator were cloned into pUC19 vector via Gibson assembly. sgRNA expression was under the control of J23119 promoter. The J23119 promoter, two BsaI recognition sites and the sgRNA scaffold were synthesized by Qingke and cloned into pUC19 vector. The lambda Red operon was cloned from pKD46 vector and placed under control of an anhydrotetracycline (aTc) inducible *P*_*tet*_ promoter, which was amplified from pBBR‐kan‐Ptet‐firefly‐GFP plasmid (Bian et al. 2022; Datsenko and Wanner 2000). The *SacB* counter‐selection cassette was amplified from p0004s plasmid and cloned into pUC19. The SpCas9 expression cassette, lambda Red operon module and *SacB* counter‐selection module were then assembled into the destination vectors with different origins of replication using the golden gate cloning method. The sgRNA expression cassette and *SacB* counter‐selection module were assembled into pUC19 to generate pUC19‐SG‐SacB plasmid. For sequential genome editing, an IPTG‐inducible sgRNA expression cassette targeting the *Rep* gene of the pBBR1 ORI was constructed. The cassette comprises the *P*_*tac*_ promoter, the *lacI* repressor gene and a sgRNA scaffold harbouring a 20‐bp spacer complementary to the *Rep* gene. The entire cassette was assembled into pRi‐Cas9‐Red‐SacB via Gibson assembly to generate the plasmid pRi‐Cas9‐Red‐SacB‐lacIsg. The sequences of the assembled plasmids were verified by the Nanopore sequencing. The construction workflow for the pBBR1‐SG‐Donor‐SacB vector was illustrated in Figure S7. Briefly, sgRNAs were designed manually. Complementary 20 nt oligonucleotides with BsaI‐compatible overhangs were synthesized by Qingke, annealed to form double‐stranded spacers and ligated into BsaI‐digested pUC19‐SG‐SacB plasmid. The sgRNA sequences are listed in Table S1. The left and right homologous arms of the repair donor were PCR amplified from the *A. tumefaciens* genome or its Ti plasmid. The primers used for donor construction are listed in Table S2. The PCR products were purified and cloned into pUC19 vector via Gibson assembly. Finally, the sgRNA expression cassette and repair donor were assembled into the pBBR1‐Spec‐CCB vector through golden gate.

### 4.3 Preparation of *A. tumefaciens* Chemical Competent Cells for Freeze–Thaw Transformation

An *A. tumefaciens* glycerol stock was streaked onto an LB agar plate and incubated at 28°C for 3 days. A single colony from the plate was inoculated into 5 mL YEB medium (5 g/L tryptone, 1 g/L yeast extract, 5 g/L beef broth, 5 g/L sucrose, 0.49 g/L MgSO_4_.7H_2_O) supplemented with 25 mg/L rifampicin and grown with shaking (280 rpm) at 28°C for 24 h. The overnight cultures were diluted by 100 mL fresh YEB medium to adjust OD_600_ to 0.1. The fresh cell cultures were then grown for 3–4 h until the OD_600_ reached 0.5. Cell cultures were chilled on ice for 10 min and harvested by centrifugation at 4000 rpm for 10 min at 4°C. The cell pellets were washed once with 10 mL precooled TE (l0 mM Tris–HCl, pH 7.5; l mM EDTA) buffer and resuspended in 1 mL ice‐cold sterile YEB medium. Aliquots of 50 μL were either used immediately for freeze–thaw transformation or flash‐frozen in liquid nitrogen and stored at −80°C. For freeze–thaw transformation, 200 ng of plasmid was mixed with 50 μL of competent cells. The mixture was incubated sequentially for 30 min on ice, 1 min in liquid nitrogen and 5 min at 37°C water bath. After incubation, 1 mL LB medium was added, and the cells were recovered by shaking at 28°C for 2 h. Finally, aliquots of 200 |μL cell cultures were plated onto LB agar plates containing appropriate antibiotics and incubated at 28°C for 2 days.

### 4.4 Preparation of *A. tumefaciens* Electrocompetent Cells for Genome Editing

Fresh electrocompetent cells of *A. tumefaciens* were prepared for each genome editing experiment. The plasmids containing Cas9 and/or lambda Red operon expression cassettes were first transformed into the *A. tumefaciens* strains GV3101 and EHA105 by the freeze–thaw method and grown at 28°C in the dark for 3 days. Then a single colony was inoculated into 3 mL LB medium supplemented with 50 mg/L kanamycin and 25 mg/L rifampicin. The overnight cultures were transferred to fresh 20 mL LB medium with antibiotics and adjusted the OD_600_ value to 0.1. The fresh culture was grown at 28°C with shaking at 280 rpm until OD_600_ reached around 0.5–0.6 (about 3 ~ 4 h). After the addition of the inducer anhydrotetracycline to a final concentration of 2 μg/mL, the cells were grown at 28°C with shaking at 280 rpm for 1 h to induce lambda Red operon expression. The cell cultures were chilled immediately on ice for 10 min and then harvested by centrifugation at 4000 rpm for 10 min at 4°C. The supernatants were discarded, and the cell pellets were washed with 10 mL ice‐cold sterile 10% glycerol two times. Finally, cells were resuspended with 250 μL of ice‐cold sterile 10% glycerol.

### 4.5 Electroporation of sgRNA and DNA Donors Into *A. tumefaciens*

The electroporation procedures for *A. tumefaciens* strains were followed with a previous study (Bian et al. 2022). For genome editing with a plasmid donor, 200 ng of donor plasmids were mixed with 40 μL electrocompetent cells. For genome editing with linear PCR donor, 200 ng of sgRNA‐containing plasmid and 1 μg PCR products were mixed with 40 μL electrocompetent cells. Electroporation was performed using 1 mm cuvettes and a Bio‐Rad MicroPulser electroporator. The mixture was transferred into an ice‐cold cuvette and electroporated at 1300 V. After electroporation, 1 mL LB medium was added into the cuvette and the cells were transferred to a fresh tube and incubated at 28°C for 3 h with shaking at 220 rpm. Then 200 μL cells were spread onto LB plates containing the appropriate antibiotics and grown at 28°C for at least 3 days to count the colonies.

### 4.6 Sequential Genome Editing and Plasmid Curing

The target sites were genotyped by PCR, and the resulting products of expected size were verified by Sanger sequencing. The primers used for genotyping are listed in Table S3. For sequential genome editing, the edited *A. tumefaciens* clones were inoculated into 3 mL LB medium supplemented with 50 μg/mL kanamycin, 25 μg/mL rifampicin and 2 mM IPTG to grow overnight at 28°C. The cultures were diluted and spread onto the LB agar plates containing kanamycin and rifampicin. Subsequently, single colonies were randomly selected to test for spectinomycin sensitivity. Colonies showing spectinomycin sensitivity indicated successful curing of pBBR1‐SG‐donor vector and could be used to prepare electrocompetent cells for the next editing round. For curing all genome editing plasmids from *A. tumefaciens*, the edited clones were inoculated into the LB‐sucrose medium without antibiotics to grow at 28°C for 24 h. The cultures were then diluted and spread onto the LB‐sucrose plate without antibiotics to grow at 28°C for 3 days. Individual colonies on the plate were streaked to test their sensitivity to kanamycin and spectinomycin. Clones exhibiting dual sensitivity to both antibiotics were considered as plasmid‐cured.

### 4.7 Genome‐Wide Off‐Target Analysis by Whole‐Genome Sequencing

To evaluate potential off‐target edits induced by the λ Red‐assisted CRISPR/Cas9 system, we performed whole‐genome sequencing of two wild type EHA105 colonies from our laboratory and twelve edited strains (post‐curing) in the EHA105 background. Genomic DNA was extracted from all strains using a bacterial genomic DNA extraction kit following the manufacturer's instructions. Whole genome sequencing was conducted on an Illumina NGS platform. Raw sequencing reads were processed and quality‐filtered using Fastp (version 0.19.7). High quality reads were mapped to the reported reference genome of EHA105 to detect single nucleotide variants (SNVs) or small insertion/deletions (indels). Three complementary variant calling tools, Genome analysis tool kit (GATK) HaplotypeCaller (v4.1.7.0), Freebayes (v0.9.21) and BCFtools mpileup (v.1.9), were used in parallel to identify SNVs or indels between mutant strains and the respective reference genomes (NCBI accession number NZ_CP067032).

### 4.8 *Agrobacterium*‐Mediated Transient Expression in *Arabidopsis* Seedlings

*Agrobacterium*‐mediated infection of *Arabidopsis* seedlings was performed using the well‐established AGROBEST assay (Wu et al. 2014). *Arabidopsis* seeds were sterilized and stratified at 4°C for 3 days and then transferred to a 6‐well plate for germination. Each well contained 10–15 seeds with 1 mL of 1/2 MS liquid medium supplemented with 0.5% (w/v) sucrose (pH 5.8). *Arabidopsis* seedlings were grown in a growth chamber at 22°C under a 16‐h/8‐h light–dark cycle. To prepare *A. tumefaciens* cell cultures for the AGROBEST infection assay, a fresh single colony was inoculated into LB medium supplemented with appropriate antibiotics and grown at 28°C for 24 h. The overnight culture was centrifuged, and the pellet was resuspended in AB‐MES medium (17.2 mM K_2_HPO_4_, 8.3 mM NaH_2_PO_4_, 18.7 mM NH_4_Cl, 2 mM KCl, 1.25 mM MgSO_4_, 100 μM CaCl_2_, 10 μM FeSO_4_, 50 mM MES, 2% glucose (w/v), pH 5.5) with 200 μM acetosyringone (without antibiotics). The culture was incubated with shaking (220 rpm) at 28°C for 16 h. Prior to infection, *A. tumefaciens* cells were pelleted by centrifugation and resuspended in fresh AB‐MES medium supplemented with 200 μM acetosyringone to an OD_600_ of 0.02. The growth medium of three‐day‐old *Arabidopsis* seedlings was replaced with 1 mL of the prepared *A. tumefaciens* suspension. Seedlings were incubated in the growth chamber for 3 days and then used for GUS staining or quantification.

### 4.9 Transient Expression in *N. benthamiana*

The binary vectors were introduced into *A. tumefaciens* via the freeze–thaw transformation method. Single colonies of *Agrobacterium* were inoculated into 5 mL LB medium supplemented with appropriate antibiotics and grew overnight at 28°C. The culture was then transferred to fresh LB medium containing the same antibiotics and 20 μM acetosyringone. The fresh culture was kept growing at 28°C until the OD_600_ reached 2.0. Cells were harvested by centrifugation and resuspended in the infiltration buffer (10 mM MgCl_2_, 10 mM MES, pH 5.6, 100 μM acetosyringone) to an OD_600_ of 0.5. The resulting suspensions were infiltrated into *N. benthamiana* leaves using a needleless syringe. Plants were maintained under a 16‐h light/8‐h dark photoperiod, and the GFP and RUBY signals were imaged 3 days post‐infiltration.

### 4.10 Quantification of GUS and RUBY Activity

GUS staining was performed as described previously (Jefferson et al. 1987). *Arabidopsis* seedlings or *N. benthamiana* leaves were harvested at the indicated time points and washed three times with cold GUS staining buffer (14 mM NaH_2_PO4, 36 mM Na_2_HPO4, 2 mM K_4_[Fe(CN)_6_], 2 mM K_3_[Fe(CN)_6_], 10% Triton). Samples were then incubated in staining buffer containing 2 mM 5‐bromo‐4‐chloro‐3‐indolyl glucuronide (X‐Gluc) under vacuum for 5 min at room temperature. Following vacuum infiltration, samples were incubated at 37°C overnight. Finally, the staining buffer was removed and samples were washed sequentially with 70% ethanol to remove chlorophyll in the tissue. GUS activity is quantified using the fluorometric substrate 4‐methylumbelliferyl‐β‐D‐glucuronide (MUG). Fluorescence of the released 4‐methylumbelliferone (4‐MU) was measured at an excitation wavelength of 365 nm and an emission wavelength of 455 nm. Relative GUS activity was calculated by normalizing the fluorescence signal to the total protein concentration, after subtracting the background signal from the mock control. The RUBY reporter promotes the synthesis of the red pigment betalain in plant tissues. Betalain production was quantified by a spectrophotometry method as previously described (Chen et al. 2023). Briefly, infiltrated leaves were harvested 3 days post‐infiltration and incubated in 100% ethanol to remove the chlorophyll pigment. Then a 0.5 cm‐diameter tissue disc was excised from the center of each infiltration site and submerged in 1 mL of sterilized water. The samples were incubated overnight at room temperature to extract betalain. Absorbance of the supernatant was measured at 538 nm wavelength using a spectrophotometer. All measurements were performed with three biological repeats.

### 4.11 Protein Extraction and Western Blot

Total proteins from infiltrated leaves were extracted using the protein extraction buffer (50 mM Tris–HCl pH 8.0, 150 mM NaCl, 10 mM MgCl_2_, 1 mM EDTA pH 8.0, 10% glycerol) supplemented with 1× Protease inhibitor cocktail (Sigma‐Aldrich). Equal amounts of total proteins from each sample were resolved by 12% SDS‐PAGE gel and then transferred onto a PVDF membrane. Membranes were blocked for 1 h at room temperature with gentle agitation in 5% skim milk dissolved in TBST buffer (20 mM Tris–HCl, pH 7.5, 150 mM NaCl and 0.1% Tween‐20). For immunoblotting, membranes were incubated 1 h at room temperature with anti‐GFP antibody (1:5000) or anti‐actin antibody (1:10000) (Sangon Biotech, Shanghai). The membranes were washed three times with TBST buffer and then blotted with HRP‐conjugated secondary antibodies. Signals were detected using High‐signal ECL Western Blotting Substrate (Abclonal) and visualized with a Quant500 imager for chemiluminescent detection with exposure times ranging from 1 s to 10 s.

## Author Contributions

K.H., Z.L. and W.H. conceived the project; M.Z. and R.L. performed the experiments; M.Z. and K.H. analysed the data; K.H. wrote the manuscript.

## Funding

This work was supported by Shanghai Landscaping and City Appearance Administration Bureau (G242402, G262402), Yuelushan Laboratory Breeding Program (YLS‐2025‐ZY04010).

## Conflicts of Interest

The authors declare no conflicts of interest.

## Supporting information


**Figure S1:** The intrinsic homologous recombination capacity of *A tumefaciens* is insufficient to repair lethal DSB. (a) Effect of the copy number of Cas9‐expression vectors on *A. tumefaciens* growth. The plates were photographed 3 days after transformation. ‘‐’ indicates non‐transformed control. (b) Number of *Agrobacterium* colonies formed on selective plates after transformation with either the empty plasmid pRi‐Cas9‐SG or the *thyA*‐targeting plasmid pRi‐Cas‐SG‐*thyA*. (c) Schematic of two‐plasmid system used to assess HR‐mediated repair of Cas9‐induced DSBs in *A. tumefacien*. (d) Colony formation on selective plate using the pRi‐Cas9 and pBBR1‐SG‐Donor two‐plasmid system for *thyA* editing. (e) PCR screening of fifteen randomly selected colonies to identify *thyA* deletion mutants. The ‘wt’ lane shows PCR amplification from wild‐type EHA105.
**Figure S2:** Efficient gene editing in EHA105 using a lambada Red recombinase‐assisted CRISPR‐Cas9 system. (a) Successful deletion of the *AmpC*, *MutS*, *MutL*, *Xse* genes using the pRi‐Cas9‐Red‐SacB and pBBR1‐SG‐Donor‐SacB two‐plasmid system. Colony PCR identified the desired deletions for each gene. (b) Schematic illustration of the M181F point mutation introduced into the *SSB* gene. Sanger sequencing chromatograms from an edited colony and a wild‐type control were shown.
**Figure S3:** Lambada Red recombinase‐assisted CRISPR/Cas9‐mediated gene deletion in *A. tumefaciens* EHA105 using linear PCR donors. (a) Schematic diagram of the pBBR1‐SG‐SacB plasmid, containing the sgRNA expression cassette and the *SacB* counter‐selection marker. (b) Design of the linear PCR donor for targeted deletion of the *thyA* gene. The homologous arm length of the donor is 300‐bp. (c) Colony formation on the selective plate following transformation with the pRi‐Cas9‐Red‐SacB, pBBR1‐SG‐SacB and the linear PCR donor for *thyA* gene. (d) PCR screening of fifteen randomly selected colonies to identify the desired *thyA* deletion mutants. (e) Design of the linear PCR donor for deletion of the *AmpC* gene. The homologous arm length of the donor is 500‐bp. (f) PCR screening of fifteen colonies to detect the desired *AmpC* deletion mutants. In panels (d) and (f), the ‘wt’ lane corresponds to PCR amplification from wild‐type EHA105.
**Figure S4:** Genome editing of *A. tumefaciens* LBA4404 strain using the modified two‐plasmid system. (a) Targeted deletion of *RecA* gene in LBA4404 using the modified two‐plasmid system, with or without aTc induction of lambada Red operon expression. Deletion events were identified by PCR screening. (b) PCR screening of colonies carrying the *thyA* deletion generated using the modified two‐plasmid system. (c) Sanger sequencing confirming the precise deletion of *thyA* gene. A representative sequencing chromatogram is shown. (d) Thymidine‐dependent growth of auxotrophic LBA4404 *thyA* mutant in liquid LB medium supplemented with or without 100 mg/L of thymidine. Wild‐type LBA4404 was used as the control. (e) Sequential deletion of *thyA* in the *RecA*‐deficient background. Both *RecA* and *thyA* deletions were verified by PCR. In panels (a), (b) and (e), ‘wt’ lane represents PCR products amplifified from wild‐type LBA4404.
**Figure S5:** Sequential multiplex gene editing of antibiotic resistance loci in EHA105. (a) Genotyping PCR results for individual deletions of the *tetR*, *strB*, *cat* and *MltB3* genes in EHA105 using the modified two‐plasmid system. (b) Sequential editing of the *strB* gene in the *tetR* mutant background. Successful double mutants were verified by PCR screening. (c) Sequential editing the *cat* gene in the *tetR‐strB* double mutant background to generate triple mutant *tetR‐strB‐cat*. (d) Final editing of the *MltB3* gene in the *tetR‐strB‐cat* triple mutant background to produce quadruple mutant *tetR‐strB‐cat‐MltB3*. The ‘wt’ lane in panel (a‐d) corresponds to PCR amplification from wild‐type EHA105.
**Figure S6:** Generation of single and double mutants of *tuf* genes in EHA105. (a) Schematic of donor design used for targeted editing of the *tufB* gene. Genotyping primers tufB out‐F and tudB in‐R amplify a diagnostic fragment to detect the editing events. For Sanger sequencing, a larger amplicon was amplified using primers tufB out‐F and tufB out‐R to span the edited site. (b) Genotyping PCR results of *tufB* edited colonies. (c) Design of the donor used for editing the *tufA* gene. Genotyping was performed using primers tufA out‐F and tudA in‐R, while the tufA out‐F and tufA out‐R primes were used for amplifying PCR products for Sanger sequencing. (d) Genotyping PCR results for *tufA* edited colonies. (e) Sequential editing of the *tufA* gene in the *tufB* mutant background to generate *tufA* ^*DC3000 4‐15*^ *tufB* ^*DC3000 4‐15*^ double mutant. Successful double mutants were verified by PCR screening and Sanger sequencing. (f) Transient GUS expression efficiency in *N. benthamiana* leaves infiltrated with EHA105, *tufA* ^*DC3000 4‐15*^, *tufB* ^*DC3000 4‐15*^ and *tufA* ^*DC3000 4‐15*^ *tufB* ^*DC3000 4‐15*^ strains. Fluorescence intensity of GUS staining reflects transient transformation efficiency. Scale bar: 0.5 cm.
**Figure S7:** Construction workflow for the pBBR1‐SG‐Donor‐SacB vector. The sgRNA spacer and repair donor template were first cloned separately into pUC19‐based vectors. Subsequently, both pUC19 constructs and the pBBR1‐Spec‐CCB destination vector were mixed and subjected to a golden gate assembly reaction to generate the final pBBR1‐SG‐Donor‐SacB vector.
**Table S1:** sgRNA sequences used in this study.
**Table S2:** Primers used for construction of donor template.
**Table S3:** Primers used for genotyping.

## Data Availability

The raw whole‐genome sequencing data reported in this study have been deposited in the National Genomics Data Center, China National Center for Bioinformation under the accession number GSA: CRA036276.
