# Supplementary material for: A Lambda Red Recombinase‐Assisted CRISPR/Cas9 System for Efficient and Precise Genome Editing in Agrobacterium tumefaciens
Source: Plant Biotechnol J. 2026 Sep 30;24(11):6010–27. doi: 10.1111/pbi.70768 (PMC13627999; doi:10.1111/pbi.70768)
Supplement: Supplementary file 1 [file PBI-24-6010-s001.docx]

**Supplemental information**

**A lambda Red recombinase-assisted CRISPR/Cas9 system for efficient and precise genome editing in *Agrobacterium tumefaciens***

Minmin Zheng^1^, Rui Liu^1^, Wei-Chang Huang^1,3^, Zuren Li^2^*, Kai Hua^1^*

1. Shanghai Key Laboratory of Plant Functional Genomics and Resources, Shanghai Chenshan Botanical Garden, and Chenshan Science Research Center, CAS Center for Excellence in Molecular Plant Sciences (CEMPS), Chinese Academy of Sciences (CAS), Shanghai 201602, China

2. Yuelushan Laboratory

3. Eastern China Conservation Centre for Wild Endangered Plant Resources, Shanghai Chenshan Botanical Garden, Shanghai 201602, China

***Corresponding author**: Zuren Li ([lizuren88214@hunaas.cn](mailto:lizuren88214@hunaas.cn)) and Kai Hua (kaihua@cemps.ac.cn)

Figure S1. The intrinsic homologous recombination capacity of *A tumefaciens* is insufficient to repair lethal DSB. (a) Effect of the copy number of Cas9-expression vectors on *A. tumefaciens* growth. The plates were photographed three days after transformation. “-” indicates non-transformed control. (b) Number of *Agrobacterium* colonies formed on selective plates after transformation with either the empty plasmid pRi-Cas9-SG or the *thyA*-targeting plasmid pRi-Cas-SG-*thyA.* (c) Schematic of two-plasmid system used to assess HR-mediated repair of Cas9-induced DSBs in *A. tumefacien*. (d) Colony formation on selective plate using the pRi-Cas9 and pBBR1-SG-Donor two-plasmid system for *thyA* editing. (e) PCR screening of fifteen randomly selected colonies to identify *thyA* deletion mutants. The “wt” lane shows PCR amplification from wild-type EHA105.

Figure S2. Efficient gene editing in EHA105 using a lambada Red recombinase-assisted CRISPR-Cas9 system. (a) Successful deletion of the *AmpC*, *MutS*, *MutL*, *Xse* genes using the pRi-Cas9-Red-SacB and pBBR1-SG-Donor-SacB two-plasmid system. Colony PCR identified the desired deletions for each gene. (b) Schematic illustration of the M181F point mutation introduced into the *SSB* gene. Sanger sequencing chromatograms from an edited colony and a wild-type control were shown.

Figure S3. Lambada Red recombinase-assisted CRISPR/Cas9-mediated gene deletion in *A. tumefaciens* EHA105 using linear PCR donors. (a) Schematic diagram of the pBBR1-SG-SacB plasmid, containing the sgRNA expression cassette and the SacB counter-selection marker. (b) Design of the linear PCR donor for targeted deletion of the *thyA* gene. The homologous arm length of the donor is 300-bp. (c) Colony formation on the selective plate following transformation with the pRi-Cas9-Red-SacB, pBBR1-SG-SacB, and the linear PCR donor for *thyA* gene. (d) PCR screening of fifteen randomly selected colonies to identify the desired *thyA* deletion mutants. (e) Design of the linear PCR donor for deletion of the *AmpC* gene. The homologous arm length of the donor is 500-bp. (f) PCR screening of fifteen colonies to detect the desired *AmpC* deletion mutants. In panels (d) and (f), the “wt” lane corresponds to PCR amplification from wild-type EHA105.

Figure S4. Genome editing of *A. tumefaciens* LBA4404 strain using the modified two-plasmid system. (a) Targeted deletion of *RecA* gene in LBA4404 using the modified two-plasmid system， with or without aTc induction of lambada Red operon expression. Deletion events were identified by PCR screening. (b) PCR screening of colonies carrying the *thyA* deletion generated using the modified two-plasmid system. (c) Sanger sequencing confirming the precise deletion of *thyA* gene. A representative sequencing chromatogram is shown. (d) Thymidine-dependent growth of auxotrophic LBA4404 *thyA* mutant in liquid LB medium supplemented with or without 100 mg/L of thymidine. Wild-type LBA4404 was used as the control. (e) Sequential deletion of *thyA* in the *RecA*-deficient background. Both *RecA* and *thyA* deletions were verified by PCR. In panels (a), (b), and (e), “wt” lane represents PCR products amplifified from wild-type LBA4404.

Figure S5. **Sequential multiplex gene editing of antibiotic resistance loci in** **EHA105**. (a) Genotyping PCR results for individual deletions of the tetR, strB, cat, and MltB3 genes in EHA105 using the modified two-plasmid system. (b) Sequential editing of the *strB* gene in the *tetR* mutant background. Successful double mutants were verified by PCR screening. (c) Sequential editing the *cat* gene in the *tetR-strB* double mutant background to generate triple mutant *tetR-strB-cat*. (d) Final editing of the *MltB3* gene in the *tetR-strB-cat* triple mutant background to produce quadruple mutant *tetR-strB-cat-MltB3*. The “wt” lane in panel (a-d) corresponds to PCR amplification from wild-type EHA105.

Figure S6. Generation of single and double mutants of *tuf* genes in EHA105. (a) Schematic of donor design used for targeted editing of the *tufB* gene. Genotyping primers tufB out-F and tudB in-R amplify a diagnostic fragment to detect the editing events. For Sanger sequencing, a larger amplicon was amplified using primers tufB out-F and tufB out-R to span the edited site. (b) Genotyping PCR results of *tufB* edited colonies. (c) Design of the donor used for editing the *tufA* gene. Genotyping was performed using primers tufA out-F and tudA in-R, while the tufA out-F and tufA out-R primes were used for amplifying PCR products for Sanger sequencing. (d) Genotyping PCR results for *tufA* edited colonies. (e) Sequential editing of the *tufA* gene in the *tufB* mutant background to generate *tufA^DC3000 4-15^ tufB^DC3000 4-15^* double mutant. Successful double mutants were verified by PCR screening and Sanger sequencing. (f) Transient GUS expression efficiency in *N. benthamiana* leaves infiltrated with EHA105, *tufA^DC3000 4-15^*, *tufB^DC3000 4-15^*, and *tufA^DC3000 4-15^ tufB^DC3000 4-15^* strains. Fluorescence intensity of GUS staining reflects transient transformation efficiency. Scale bar: 0.5 cm.

Figure S7. Construction workflow for the pBBR1-SG-Donor-SacB vector. The sgRNA spacer and repair donor template were first cloned separately into pUC19-based vectors. Subsequently, both pUC19 constructs and the pBBR1-Spec-CCB destination vector were mixed and subjected to a golden gate assembly reaction to generate the final pBBR1-SG-Donor-SacB vector.

Table S1. sgRNA sequences used in this study

| *Agrobacterium* strain | Target gene | sgRNA sequence |
| --- | --- | --- |
| EHA105/GV3101 | *thyA* | CGGCTCACTCATACCGCGAT |
| EHA105/GV3101 | *RecA* | GGCGACGACGGCAGCGACGA |
| EHA105 | *rpoB* | AGCCGAAAGACGGCGCTTGA |
| GV3101 | *rpoB* | TCGGTTCCTCGCAGCTGTCG |
| EHA105/GV3101 | *AmpC* | CGCAGAGACGCGTTCCTCAT |
| EHA105 | *MutS* | GGAGCAATATATCGAGATCA |
| EHA105 | *MutL* | TTCAATGTAGGCGTCAGCTG |
| EHA105 | *SSB* | CGGTGGCTTCTCCAACGATA |
| EHA105/GV3101 | *Xse* | AAGATTGCTCAAGGCGGTGT |
| EHA105 | *cat* | GGGCATTACACTCGACAAGC |
| EHA105 | *MltB3* | TTGCATGATGTTTGCAGGCT |
| EHA105 | *strB* | TCCGGCCTCGTGGAATATCG |
| EHA105 | *TetR* | TTAAGCCGCCACAGCCTTCG |
| EHA105 | *tufA* | ACTTGCTCTTTGCCATTAGT |
| EHA105 | *tufB* | TTCACAGGAAGCAATTCACA |
| EHA105 | T-DNA left border | TTACCGCACCTCTGCAGCAG |
| GV3101 | *VirD2* (for insertion) | CGATCGGGCATCGAATATGT |
| GV3101 | *VirD2* (for deletion) | AGGACGGGCGTGGGGGAAAT |
| LBA4404 | *RecA* | CGATCTTCTCCGGCATGTGA |
| LBA4404 | *thyA* | TAACCTAAAGGTGGATCGGA |

Table S2. Primers used for construction of donor template

| Primer name | Primer sequence (5′-3′) | Purpose |
| --- | --- | --- |
| thyA-LA300-F | *CGACGGCCAGTGAATTC*CACCTGCCTGGTGAG CACGCCCAGTTCCACAGCCTTCGT | Amplifying 300-bp left HA for *thyA* deletion |
| thyA-LA300-R | ATGAGTGAGCCGCGCGTCACCATC |  |
| thyA-RA300-F | GACGCGCGGCTCACTCATGCGTTCTGCCGCCGATTCCCTGAT | Amplifying 300-bp left HA for *thyA* deletion |
| thyA-RA300-R | *CATGATTACGCCAAGCTT*CACCTGCCTCGAGCG GGATTCGTCTCTGGTGTCCGCTTC |  |
| thyA-LA50-F | *CGACGGCCAGTGAATTC*CACCTGCCTGGTGAG ACGCCGTTCTCGGAGACCGCAAC | Amplifying 50-bp HAs for *thyA* deletion |
| thyA-RA50-R | *CATGATTACGCCAAGCTT*CACCTGCCTCGAGCG GCCCTGTGCTATTATATTCCCTT |  |
| thyA-LA100-F | *CGACGGCCAGTGAATTC*CACCTGCCTGGTGAG GCTTCAGATCGGTCGACAGCTTC | Amplifying 100-bp HAs for *thyA* deletion |
| thyA-RA100-R | *CATGATTACGCCAAGCTT*CACCTGCCTCGAGCG CGCTTTGATCTGAGCCGGGGATA |  |
| thyA-LA500-F | *CGACGGCCAGTGAATTC*CACCTGCCTGGTGAG GTGGTAAACAGAACCGGATAATTG | Amplifying 500-bp HAs for *thyA* deletion |
| thyA-RA500-R | *CATGATTACGCCAAGCTT*CACCTGCCTCGAGCG TAATTTAGACTATTCTTGACGAC |  |
| thyA-LA800-F | *CGACGGCCAGTGAATTC*CACCTGCCTGGTGAG ATTGCCGCCGCCACCGCCGCGAG | Amplifying 800-bp HAs for *thyA* deletion |
| thyA-RA800-R | *CATGATTACGCCAAGCTT*CACCTGCCTCGAGCG GTTGTTGTCATTTGCAACTACAC |  |
| thyA-LA1000-F | *CGACGGCCAGTGAATTC*CACCTGCCTGGTGAG ATTTCGTCCTTCGGACGGCCGAA | Amplifying 1000-bp HAs for *thyA* deletion |
| thyA-RA1000-R | *CATGATTACGCCAAGCTT*CACCTGCCTCGAGCG TACGCAATGTCCCGAACGGCAGCA |  |
| RecA-LA-F | *CGACGGCCAGTGAATTC*CACCTGCCTGGTGAG GCACTATCTCGTGTCCGTTCTTC | Amplifying left HA for *RecA* deletion |
| RecA-LA-R | TAGATTCCGCGAAGGTCCGGGCAT |  |
| RecA-RA-F | GGACCTTCGCGGAATCTACCGAACCACCTTTAGGTTATTGAAG | Amplifying right HA for *RecA* deletion |
| RecA-RA-R | *CATGATTACGCCAAGCTT*CACCTGCCTCGAGCG GATCATGCCGCGCCTGTCGATAT |  |
| RecA-apa-LA-F | *CGACGGCCAGTGAATTC*CACCTGCCTGGTGAG GATCATGCCGCGCCTGTCGATATGC | Amplifying left HA for *RecA* replacement |
| RecA-apa-LA-R | CCGAACCACCTTTAGGTTATTG |  |
| Apramycin-F | CCTAAAGGTGGTTCGGATGTCATCAGCGGTGGAGT | Amplifying apramycin resistance gene |
| Apramycin-R | ACCTTCGCGGAATCTATCAGCCAATCGACTGGCGAGC |  |
| RecA-apa-RA-F | TAGATTCCGCGAAGGTCCGGGCATC | Amplifying right HA for *RecA* replacement |
| RecA-apa-RA-R | *CATGATTACGCCAAGCTT*CACCTGCCTCGAGCG GGCACTATCTCGTGTCCGTTCTTCT |  |
| 105rpoB-LA-F | *CGACGGCCAGTGAATTC*CACCTGCCTGGTGAG CCAGGAAGCGCTGTTCGACATC | Amplifying left HA for EHA105 *rpoB* substitution |
| 105rpoB-LA-R | GCGTGATTTCCGAAAGCGGGTTCACC |  |
| 105rpoB-RA-F | CGCTTTCGGAAATCACGCACAAGCGCCGTCTTTCGGCTCTTGGAC | Amplifying right HA for EHA105 *rpoB* substitution |
| 105rpoB-RA-R | *CATGATTACGCCAAGCTT*CACCTGCCTCGAGCG AAGCGGAACGGCCTGACGCTGCA |  |
| 3101rpoB-LA-F | *CGACGGCCAGTGAATTC*CACCTGCCTGGTGAG ATATCCGCAACACGCTGTCTGCGG | Amplifying left HA for GV3101 *rpoB* substitution |
| 3101rpoB-LA-R | CTGGCTCAGCTGCGAGGAACCGAAGAA |  |
| 3101rpoB-RA-F | TCCTCGCAGCTGAGCCAGTTCATGGACCAGGTGAACCCGCTTTC | Amplifying right HA for GV3101 *rpoB* substitution |
| 3101rpoB-RA-R | *CATGATTACGCCAAGCTT*CACCTGCCTCGAGCG GCGCGGTTGGCGTCGTCGTTTTCC |  |
| AmpC-LA-F | *CGACGGCCAGTGAATTC*CACCTGCCTGGTGAG TAACTCGGCACACGCCCACCGACG | Amplifying left HA for *AmpC* deletion |
| AmpC-LA-R | TAACCCTGTCCGCCGTTTGATGT |  |
| AmpC-RA-F | AAACGGCGGACAGGGTTACATCTTTCGTGTTCTCCAAAACCTA | Amplifying right HA for *AmpC* deletion |
| AmpC-RA-R | *CATGATTACGCCAAGCTT*CACCTGCCTCGAGCG CGGATGCCGCTTTTGAAATGCCGC |  |
| MltB3-LA-F | *CGACGGCCAGTGAATTC*CACCTGCCTGGTGAG CCCGCCTTGGGCAGAAGGCCGGCC | Amplifying left HA for *MltB3* deletion |
| MltB3-LA-R | CATCGTCAGTCTTTCGTTCTCTCG |  |
| MltB3-RA-F | AACGAAAGACTGACGATGTGATAATGTGCCGGCGAATTCGGC | Amplifying right HA for *MltB3* deletion |
| MltB3-RA-R | *CATGATTACGCCAAGCTT*CACCTGCCTCGAGCG GAACCGTTGGCACGCACATCGACG |  |
| MutS-LA-F | *CGACGGCCAGTGAATTC*CACCTGCCTGGTGAG TTGATCATGCTTTCAACACCACCG | Amplifying left HA for *MutS* deletion |
| MutS-LA-R | CGGAGTGGCCGTCGCGCGGCTCTC |  |
| MutS-RA-F | CGCGCGACGGCCACTCCGAGAGATACAGTGCCTGTCCATCGC | Amplifying right HA for *MutS* deletion |
| MutS-RA-R | *CATGATTACGCCAAGCTT*CACCTGCCTCGAGCG GGCGCAATCAATGCCGCTGCCATC |  |
| MutL-LA-F | *CGACGGCCAGTGAATTC*CACCTGCCTGGTGAG GCCACGCTCCAGTAGCGCAGCTC | Amplifying left HA for *MutL* deletion |
| MutL-LA-R | CGCCTACATTGAATGGCAAGGCA |  |
| MutL-RA-F | TGCCATTCAATGTAGGCGGATCGTCATTTTCGCGGATTCGCTGC | Amplifying right HA for *MutL* deletion |
| MutL-RA-R | *CATGATTACGCCAAGCTT*CACCTGCCTCGAGCG CCGGGATGAAACGACAAATTCCGA |  |
| SSB-LA-F | *CGACGGCCAGTGAATTC*CACCTGCCTGGTGAG GTCGTCGAACAATATGTCAAGAAG | Amplifying left HA for *SSB* substitution |
| SSB-LA-R | AAAATCGTTGGAGAAGCCACCGGAA |  |
| SSB-RA-F | GGCTTCTCCAACGATTTTGATGACGATATTCCGTTCTGAG | Amplifying right HA for *SSB* substitution |
| SSB-RA-R | *CATGATTACGCCAAGCTT*CACCTGCCTCGAGCG ACCACTGGCGCGGGACTCCGCAAC |  |
| Xse-LA-F | *CGACGGCCAGTGAATTC*CACCTGCCTGGTGAG CCATCGCGACACCGTAGGCGCCG | Amplifying left HA for *Xse* deletion |
| Xse-LA-R | GACCGTTTGTAACCAAAAGCCGTC |  |
| Xse-RA-F | TTTGGTTACAAACGGTCTCAGATCGACGCCGCATCCGAA | Amplifying right HA for *Xse* deletion |
| Xse-RA-R | *CATGATTACGCCAAGCTT*CACCTGCCTCGAGCG TGCCGGTGGCGCCGCACGATTGC |  |
| TetR-LA-F | *CGACGGCCAGTGAATTC*CACCTGCCTGGTGAG CAATGTACCGTCAGACCGGTCGCC | Amplifying left HA for *TetR* deletion |
| TetR-LA-R | TAAAGACTCCGGACGTCATGCGCC |  |
| TetR-RA-F | TGACGTCCGGAGTCTTTATAGGCGAAGACTCTGGCGCGGACG | Amplifying right HA for *TetR* deletion |
| TetR-RA-R | *CATGATTACGCCAAGCTT*CACCTGCCTCGAGCG TGTTTGGGCTCAATGTCGTGGGTG |  |
| strB-LA-F | *CGACGGCCAGTGAATTC*CACCTGCCTGGTGAG GTCTCCGGTTACACCGGCGGTGGC | Amplifying left HA for *strB* deletion |
| strB-LA-R | GATCATGCCGAGAGCATCAACTCC |  |
| strB-RA-F | GATGCTCTCGGCATGATCTGAGCGGGGTCAGTCCCTGACTTC | Amplifying right HA for *strB* deletion |
| strB-RA-R | *CATGATTACGCCAAGCTT*CACCTGCCTCGAGCG GAGCGGACTGGCAGGGGCTTTGCCG |  |
| cat-LA-F | *CGACGGCCAGTGAATTC*CACCTGCCTGGTGAG CCGGTGTTCTGCTTTTTACGCTGCG | Amplifying left HA for *cat* deletion |
| cat-LA-R | TGAAATAATCTCACTGCATCTGTT |  |
| cat-RA-F | GCAGTGAGATTATTTCAATGCTCTAGCGGTAAAGATTGCCGGCCC | Amplifying right HA for *cat* deletion |
| cat-RA-R | *CATGATTACGCCAAGCTT*CACCTGCCTCGAGCG GGTTACTATATCGATGTTGGTGCC |  |
| tufA-LA-F | *CGACGGCCAGTGAATTC*CACCTGCCTGGTGAG GGTGACGTGATCGGCGATCTG | Amplifying left HA for *tufA* substitution |
| tufA-LA-R | TGCGGTCAAACTTCTCCTTTGCCATTGATGGCTCTCCATTCTTGTCCCTT |  |
| tufA-RA-F | GAGAAGTTTGACCGCAGTTTGCCGCACTGCAACGTTGGCACGATCGGTCACGTCG | Amplifying right HA for *tufA* substitution |
| tufA-RA-R | *CATGATTACGCCAAGCTT*CACCTGCCTCGAGCG GAGCAAACCAGGATCGCGCCGT |  |
| tufB-LA-F | *CGACGGCCAGTGAATTC*CACCTGCCTGGTGAG TCCCCCGGCGATGGAATGTCCT | Amplifying left HA for *tufB* substitution |
| tufB-LA-R | ACTGCGGTCAAACTTCTCCTTTGCCATGCAAATTGCTTCCTGTGAACGTGAAGTC |  |
| tufB-RA-F | GAGAAGTTTGACCGCAGTTTGCCGCACTGCAACGTTGGCACGATCGGTCACGTCGAC | Amplifying right HA for *tufB* substitution |
| tufB-RA-R | *CATGATTACGCCAAGCTT*CACCTGCCTCGAGCG CCGTCGGCAGCCGAGCAAACCAG |  |
| EHA105T-LA-F | *CGACGGCCAGTGAATTC*CACCTGCCTGGTGAG GATCGACCGCGTCATGGC | Amplifying left HA for 105 T-DNA replacement |
| EHA105T-LA-R | ACAAATAGGGGTTCCGCGGTTGCTGCAGAGGTGCGGTAAGT |  |
| GenR-F | CGCGGAACCCCTATTTGT | Amplifying gentamicin resistance gene |
| GenR-R | CTGAATTCCGCAGAAAGG |  |
| EHA105T-RA-F | CCTTTCTGCGGAATTCAGATGTCCATTCATCCATCCA | Amplifying right HA for 105 T-DNA replacement |
| EHA105T-RA-R | *CATGATTACGCCAAGCTT*CACCTGCCTCGAGCG GCGCCGCGGATTTGATGA |  |
| VirD2-W-LA-F | *CGACGGCCAGTGAATTC*CACCTGCCTGGTGAG AGCGTCCTCATGATGACGACGGA | Amplifying left HA for VirD2 omega domain deletion |
| VirD2-W-LA-R | TGCTGCGCTCATCTCTCTCGCG |  |
| VirD2-W-RA-F | AGAGAGATGAGCGCAGCAAGAATAGGAGATAGAGAAGGACCGAGTGA | Amplifying right HA for VirD2 omega domain deletion |
| VirD2-W-RA-R | *CATGATTACGCCAAGCTT*CACCTGCCTCGAGCG GGTCCGTGCGGGCTTCGGATATTC |  |
| VirD2-N-LA-F | *CGACGGCCAGTGAATTC*CACCTGCCTGGTGAG GTGCGCCGCATTGGTGGCTT | Amplifying left HA for tag insertion at N-terminus of VirD2 |
| VirD2-N-LA-R | ACCATATGGTCACGGGGCATCGAATATGTTCCCCGCCAAGT |  |
| GFP_11_-3HA-F1 | ATGCCCCGTGACCATATGGTTCTTCATGAGTACGTAAATGCTGCTGGCATCACA | Amplifying GFP_11_-3HA tag sequence |
| GFP_11_-3HA-F | AATGCTGCTGGCATCACAAGCGGCGGCAGCTACCCATACGATGTTCCAGA |  |
| GFP_11_-3HA-R | ACTTGAGCTCGATCACCCCTGGCATAGTCGGGGACATCAT |  |
| VirD2-N-RA-F | GATCGAGCTCAAGTTATCA | Amplifying right HA for tag insertion at N-terminus of VirD2 |
| VirD2-N-RA-R | *CATGATTACGCCAAGCTT*CACCTGCCTCGAGCG CGCATAAGCCGCTACCTGGC |  |
| 4404-RecA-LA-F | *CGACGGCCAGTGAATTC*CACCTGCCTGGTGAG AAAACGAAGGGCGTATCTTCCACGC | Amplifying left HA for LBA4404 *RecA* deletion |
| 4404-RecA-LA-R | CCGATCCACCTTTAGGTTATTGAA |  |
| 4404-RecA-RA-F | CCTAAAGGTGGATCGGTGCATTCGAAGGTCCGGGCGTTC | Amplifying right HA for LBA4404 *RecA* deletion |
| 4404-RecA-RA-R | *CATGATTACGCCAAGCTT*CACCTGCCTCGAGCG GAAGAACGTATGGTGACGGGCCGT |  |
| 4404-thyA-LA-F | *CGACGGCCAGTGAATTC*CACCTGCCTGGTGAG TAGACAATTCTTGACGACGAGGGA | Amplifying left HA for LBA4404 *thyA* deletion |
| 4404-thyA-LA-R | CATGTGTTCTGCCGCCGATTCCCT |  |
| 4404-thyA-RA-F | GGCGGCAGAACACATGTGAGCGAGCCGCGCATCACCATCA | Amplifying right HA for LBA4404 *thyA* deletion |
| 4404-thyA-RA-R | *CATGATTACGCCAAGCTT*CACCTGCCTCGAGCG AGTGGTGAACAGAACCGGATAATT |  |

Note: The AarI recognition sites are underlined. Letters in italic indicate the homologous arms designed for Gibson assembly into pUC19 vector.

Table S3. Primers used for genotyping

| Primer name | Primer sequence (5′-3′) | Purpose |
| --- | --- | --- |
| thyA-GT-300bp-F | GGCATGGCCTGACGGTAAATCTCG | Screening the 50-bp, 100-bp, and 300-bp deletion events at *thyA* site |
| thyA-GT-300bp-R | TCGTCAAACATCTCGGCATTGCCC |  |
| RecA-GT-F | CGGCATTGGTGAACATCAGTGTCG | Screening the deletion and replacement events at *RecA* site |
| RecA-GT-R | TCTTCCATCCACGAGCCCGGATTG |  |
| thyA-GT-1000bp-F | AGATGGAAATGCAGGCCAGGCATC | Screening the 500-bp, 800-bp, and 1000-bp deletion events at *thyA* site |
| thyA-GT-1000bp-R | ATTTTCGATCCTGACGGCTACGCG |  |
| rpoB-GT-F | TCCGGCTTTGACGAGATTCCGGTAC | PCR amplifying and sequencing the target sites in EHA105 and GV3101 *rpoB* gene |
| rpoB-GT-R | CAGAAGCGGAACGGCCTGACGCTGC |  |
| AmpC-GT-F | GGACCGATAAAAGCGAAGGACGAG | Screening the deletion events at *AmpC* site |
| AmpC-GT-R | CGGTTGTTGTTGGTGGAAACCCTG |  |
| MltB3-GT-F | CACCTGCTGGCGCGTGAAGCTTGC | Screening the deletion events at *MltB3* site |
| MltB3-GT-R | GGCGAACCAGTCGTAATAATCCTT |  |
| MutS-GT-F | CTCTATCATGAAATGGACCTCGTG | Screening the deletion events at *MutS* site |
| MutS-GT-R | ACCGCGATCTGCGGCGCATCCTTG |  |
| MutL-GT-F | CCGCCGCGTCCACATAGGCCTCCT | Screening the deletion events at *MutL* site |
| MutL-GT-R | CTATTGGAATCGTCCAGGGCAGAT |  |
| SSB-GT-F | GACCGAATGGCACACCGTCGTGG | PCR amplifying and sequencing the target site in *SSB* gene |
| SSB-GT-R | GCTACACGTTCAGCTTCCGCGAAC |  |
| Xse-GT-F | GCTGCAGCACCAGAAGGCCGATCA | Screening the deletion events at *Xse* site |
| Xse-GT-R | CTGGGCTTTCCGCGATCTCCTGG |  |
| TetR-GT-F | AGACCTGGCTGAATTTTGCGCGGG | Screening the deletion events at *TetR* site |
| TetR-GT-R | GCGCCTTCATTTCGGCTGTTCACGC |  |
| strB-GT-F | CGCTAGCGGCATCCTGCCCGACGGT | Screening the deletion events at *strB* site |
| strB-GT-R | GGCTGCATGTGGATATTGCGAGGC |  |
| cat-GT-F | GCTTCCGGCAGGGCGCTGAGGGCA | Screening the deletion events at *cat* site |
| cat-GT-R | GCTCGACTTCGGCGATGACGAGTC |  |
| tufA Out-F | GAGCCGATGATGAAGGTCGAAGTC | PCR amplifying and sequencing the target site in *tufA* gene |
| tufA Out-R | GGAACGCCAACCTGACGGGCAAGC |  |
| tufA in-R | AAACTGCGGTCAAACTTCTC | tufA Out-F and tufA in-R were used for detecting the substitution events in *tufA* gene |
| tufB Out-F | GCGAAGGATCGATACGGAGCTCGG | PCR amplifying and sequencing the target site in *tufB* gene |
| tufB Out-R | CCTGACGGGCAAGCAGGATGTGCT |  |
| tufB in-R | AAACTGCGGTCAAACTTCTC | tufB Out-F and tufB in-R were used for detecting the substitution events in *tufB* gene |
| EHA105T-GT-F | GTCAGCGATGGCAGTGAAGATGGC | Screening the replacement events at EHA105 T-DNA border sequence |
| EHA105T-GT-R | GACGGCGGCGTCGAACATGTTGTGG |  |
| 3101-omega-GT-F | GACACACGACCGGCAGCGGCGTG | PCR amplifying and sequencing the omega domain of GV3101 *VirD2* gene |
| 3101-omega-GT-R | CAAGCCCACGCAGATACTGCATAC |  |
| 3101-GFP_11_-GT-F | CTCTCCGACAGCATGGCCATACGG | Screening the insertion events at the N-terminus of GV3101 *VirD2* gene |
| 3101-GFP_11_-GT-R | CATCTCGGCTGCCCACTCCCGGC |  |
| 4404-RecA-GT-F | TCCAGAATGGCGGTGTAGCTTGG | Screening the deletion events at LBA4404 *RecA* site |
| 4404-RecA-GT-R | CATGGGTAATCGCCTCTTCCTTGA |  |
| 4404-thyA-GT-F | CATGAAAAGATTTCGTGTTTGCCGCC | Screening the deletion events at LBA4404 *thyA* site |
| 4404-thyA-GT-R | CAGGGTAAATAGTTCACTTTGACG |  |
